# Research Progress on the Structure and Properties of Medical Bone Implants Based on Additive Manufacturing

**DOI:** 10.3390/mi17091043

**Published:** 2026-08-31

**Authors:** Hongtao Yu, Yanquan Tong, Bingwei Gao, Feng Wang

**Affiliations:** 1 Department of Radiology, 242 Hospital, Harbin 150066, China; wangfeng19941997@163.com; 2 School of Mechanical and Power Engineering, Harbin University of Science and Technology, Harbin 150080, China; tongyanquan223@163.com (Y.T.); gaobingwei@hrbust.edu.cn (B.G.)

**Keywords:** additive manufacturing, bone implants, porous structures, mechanical properties, osseointegration

## Abstract

Conventional medical bone implants exhibit severe mechanical mismatch with native bone, which easily induces adverse outcomes including stress shielding and implant loosening. Additive manufacturing (AM) provides a novel strategy to fabricate patient-specific implants with complex geometric architectures. Based on the additive manufacturing of medical bone implants, this paper summarizes the design criteria, structural types, design methods and performance control mechanisms, and deeply analyzes the influence mechanism of structural design on elastic modulus matching, fatigue performance, osseointegration and wear resistance. The research shows that the elastic modulus of implants can be effectively matched to that of native bone and osseointegration can be promoted by accurately regulating the porosity, pore size and topological configuration. The present work provides theoretical guidance for the structural optimization design and clinical translation of high-performance bone implants.

## 1. Introduction

Bone serves as the primary load-bearing structure of the human locomotor system, and its structural integrity is critical for sustaining normal physiological functions. However, bone defects caused by trauma, tumor resection, osteoporosis and other factors not only seriously damage the motor function and quality of life of patients, but also impose stringent demands on clinical orthopedic repair techniques [1,2,3,4]. In the long-term application of traditional bone implants, it has been found that there is a significant difference between the elastic modulus of dense metal implants represented by Ti-6Al-4V alloy and that of human cortical bone. The imbalance of mechanical properties between the implant and the host bone will directly lead to implant loosening or even secondary fracture, which will seriously affect the therapeutic effect [5,6]. Meanwhile, implants prepared by traditional manufacturing technology often have problems such as insufficient interface fit and poor initial stability in complex bone defect repair cases, which further affect the clinical treatment effect [7]. With the rapid development of additive manufacturing technology, a new solution is provided to overcome the above problems. Powder Bed Fusion, represented by selective laser melting and electron beam melting, breaks through the geometric constraints of traditional subtractive manufacturing and casting molding, can fabricate patient-specific implants with complex geometric shapes in a single molding process, and realizes precise regulation of the overall mechanical properties through the design of an internal porous structure [8,9]. The equivalent elastic modulus of the implant can be controlled within a certain range by adjusting the porosity and pore size. Meanwhile, interconnected porous structures can provide space for cell adhesion, proliferation and nutrient transport, and create a beneficial microenvironment for osseointegration [10,11,12].

Although there are a large number of literature reviews on the manufacture of bone implants with additives in this field, most of them only focus on a single structural type or emphasize mechanical properties while ignoring biological response, and work on systematically and critically integrating structural design selection with the induced coupling of mechanical and biological properties is still obviously insufficient. In particular, wear resistance is a key factor affecting the long-term stability of joint-bearing implants, and the existing reviews rarely discuss it systematically from the perspective of structural design. This review broadens the perspective to the whole system level, and regards mechanical matching, fatigue durability, wear resistance and osseointegration as interdependent design goals rather than isolated performance indicators. Meanwhile, the current academic disputes in this field are summarized, and the applicable clinical scenarios of different structural schemes are clarified. Based on the above research background, this paper focuses on additive manufacturing, medical bone implants, structural design and mechanical properties. According to the selection principles of relevance, novelty and academic quality, the published related literature is systematically reviewed and analyzed, and original research papers and systematic reviews focusing on the structural design, mechanical regulation and biological performance of additive manufacturing medical bone implants are screened. The referenced studies need to have reliable experimental data or complete mechanism analysis. At the same time, papers with incomplete data, obvious logical loopholes and repeated publications are excluded. On this basis, this paper systematically summarizes the mainstream structural design methods for manufacturing medical bone implants with additive manufacturing over the past five years, and the influence mechanisms of structural parameters on mechanical and biological properties. Firstly, the core design principles and key structural parameters of bone implants are expounded, and then the typical structural types and design methods of bone implants are summarized. In the end, the regulatory effect of structural design on elastic modulus matching, fatigue performance, osseointegration and wear resistance is analyzed, which provides theoretical reference and research ideas for the structural optimization design and clinical translation application of high-performance bone implants to meet clinical needs.

## 2. Structural Design Criteria and Biological Requirements of Bone Implants

The rapid development of additive manufacturing technology provides huge innovation space for bone implant design, but it also necessitates more rigorous requirements for the design process. Unlike traditional manufacturing with reduced materials, the design of bone implants with additive manufacturing must take into account three aspects: biomechanical adaptation, osseointegration and biocompatibility, and process manufacturability. Thus, the establishment of systematic design criteria is the premise of manufacturing high-performance bone implants.

### 2.1. Basic Principles of Design

#### 2.1.1. Biomechanical Adaptation Principle

Host bones have the ability for dynamic reconstruction, and their density and structure will be adjusted with a change in the mechanical environment. When the stiffness of the implant is too high, it will cause stress shielding in the surrounding bone tissue, which will lead to bone resorption and implant loosening [13,14]. The elastic modulus of traditional metal implant materials is as high as 100–120 GPa, far exceeding the stiffness range of native implants. Therefore, reducing the overall elastic modulus through a porous structure is an effective method to avoid stress shielding [15,16]. The elastic modulus of porous structures is also affected by topological structure and relative density.

**Figure 1:**
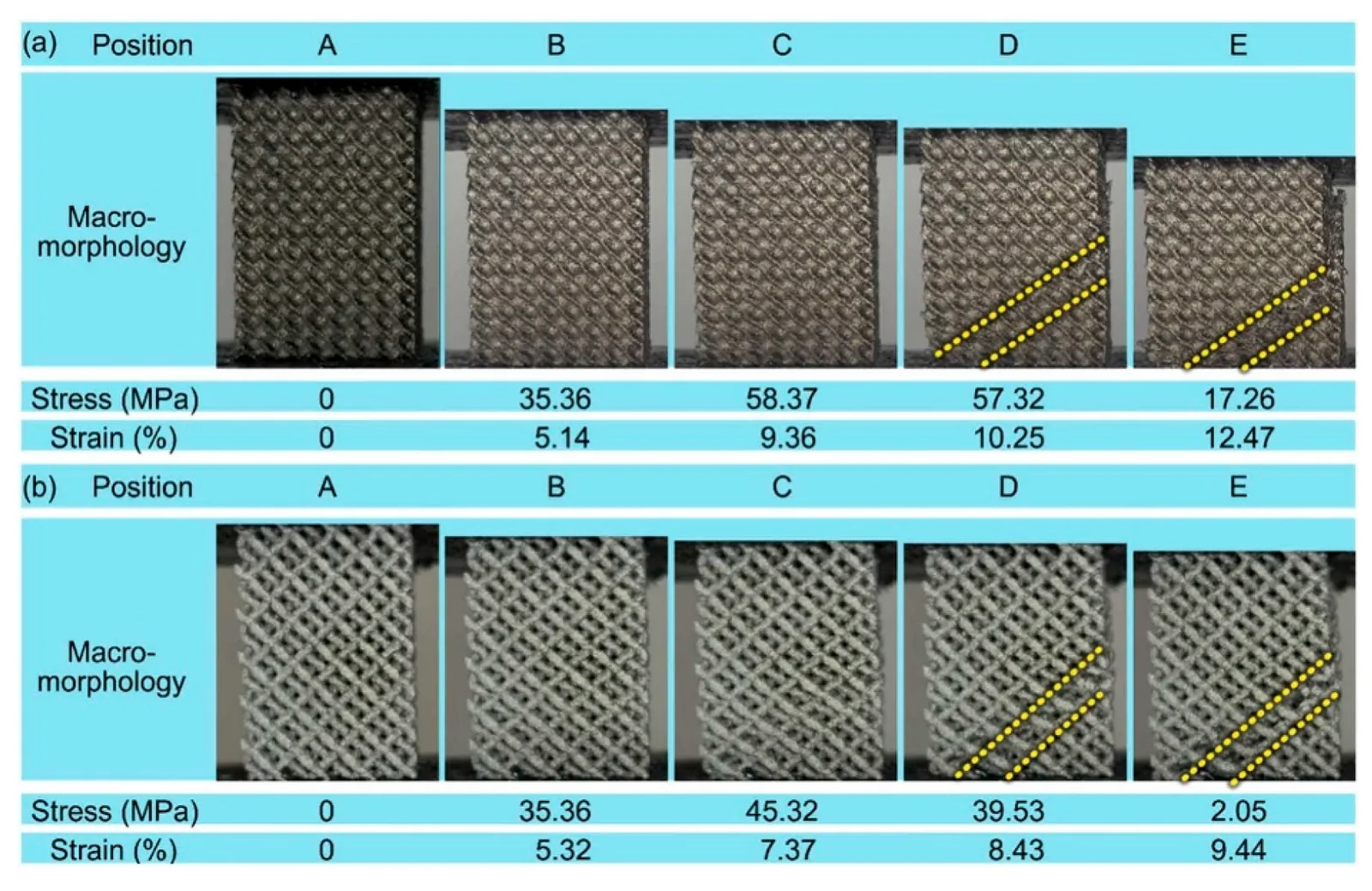
Video snapshots captured during the compressive test: (**a**) the TPMS-D structure and (**b**) the Strut-D structure [17].

Zhang et al. [17] compared the deformation and compression performance of Strut-D and TPMS-D structures based on numerical simulation and compression experiments, and found that the elastic modulus of the TPMS-D structure was 18% higher than that of the Strut-D structure. The reason is that the continuous curved surface of the TPMS-D structure makes the stress distribution more uniform, and avoids stress concentration at strut nodes. At the same time, it shows that the TPMS-D structure is particularly beneficial to the application of bone implants under impact load. Figure 1 compares the deformation processes of TPMS-D (Figure 1a) and Strut-D (Figure 1b) through video snapshots, which further proves that the TPMS-D structure with uniform stress distribution shows higher initial compressive strength. In the application of spinal fusion, Mehboob [18] found through finite element analysis that the combination of carbon fiber-reinforced PEEK rods with low stiffness and a PEEK cage can transfer more load to bone implants and reduce stress shielding, while providing near-natural intervertebral micro-motion, thus reducing the risk of degeneration of adjacent segments. Fatigue performance is also an important part of biomechanical adaptation. Gandhi et al. [19] systematically summarized the fatigue behavior of lattice structures made by additive manufacturing, and pointed out that the high-cycle fatigue strength of a TPMS structure, especially a Gyroid structure, is much better than that of a pillar structure because of its continuous and smooth surface.

#### 2.1.2. Principles of Osseointegration and Biocompatibility

Osseointegration refers to the formation of a direct, stable and functional connection between the implant and the host bone without fibrous tissue intervention [20]. Biocompatibility demands that the implant does not cause immune rejection, inflammation or cytotoxic reaction in the physiological environment, and can support the normal adhesion, proliferation and differentiation of cells. Both of them constitute the biological basis of the structural design of bone implants. The biocompatibility and osseointegration of β-tricalcium phosphate (β-TCP) ceramics prepared by 3D printing with SLA were systematically studied by Li et al. [21], and in vitro experiments were carried out in comparison with commonly used clinical materials. The experimental results showed that SLA-molded β-TCP not only had no hemolytic toxicity, but its hydrophilic surface and porous structure also significantly promoted the adhesion, proliferation and differentiation of osteoblasts, which provided an important experimental basis for the material selection and structural design of bone implants. Jacobs et al. [22] compared the biological performance of two Ti-6Al-4V alloy implants through a rabbit femoral implant model, and found that the surface roughness directly affected the adhesion, growth and proliferation of osteoblasts, and the roughened titanium implant could significantly improve the osseointegration effect compared with the smooth surface. Wang Jie and Li Haopeng [23] summarized four main design paths of surface modification: sandblasting and acid etching, dealloying, micro-arc oxidation and plasma spraying. Although they are different in technical principles, their main purposes are to establish a microenvironment conducive to protein adhesion, cell response and bone tissue growth on the surface of materials. Yan et al. [24] modified Ti-6Al-4V by poly-dopamine/copper composite coating and treated Escherichia coli and Staphylococcus aureus with 808 nm laser irradiation. The study found that osteoblasts adhered and stretched well on the surface of the material, and no cytotoxicity was detected. The work provides a feasible method for the surface modification of medical titanium alloys to achieve controllable antibacterial properties and biocompatibility.

Through a preliminary study on the mechanism of osseointegration at the level of histology and cytology, Jia Peng and Zhang Tao [25] summarized the characteristics of interbody osseointegration of biological cages, and pointed out that the combination of biphasic or multiphase materials could make up for the deficiencies of mechanics and bioactivity, and the curvature and placement position of the cage were important factors affecting osseointegration. Lang Wei [26] pointed out from the perspective of medical engineering that a porosity of 60–80% could not only ensure sufficient strength but also be beneficial to vascularization, and a pore size of 200–600 μm was suitable for bone tissue growth. It is a universal conclusion formed by current research, and its optimal value under specific implantation site and load conditions still lacks sufficient clinical verification, so it cannot be directly used as a general design rule applicable to all clinical scenarios.

#### 2.1.3. Adaptability Principle of Additive Manufacturing Process

**Figure 2:**
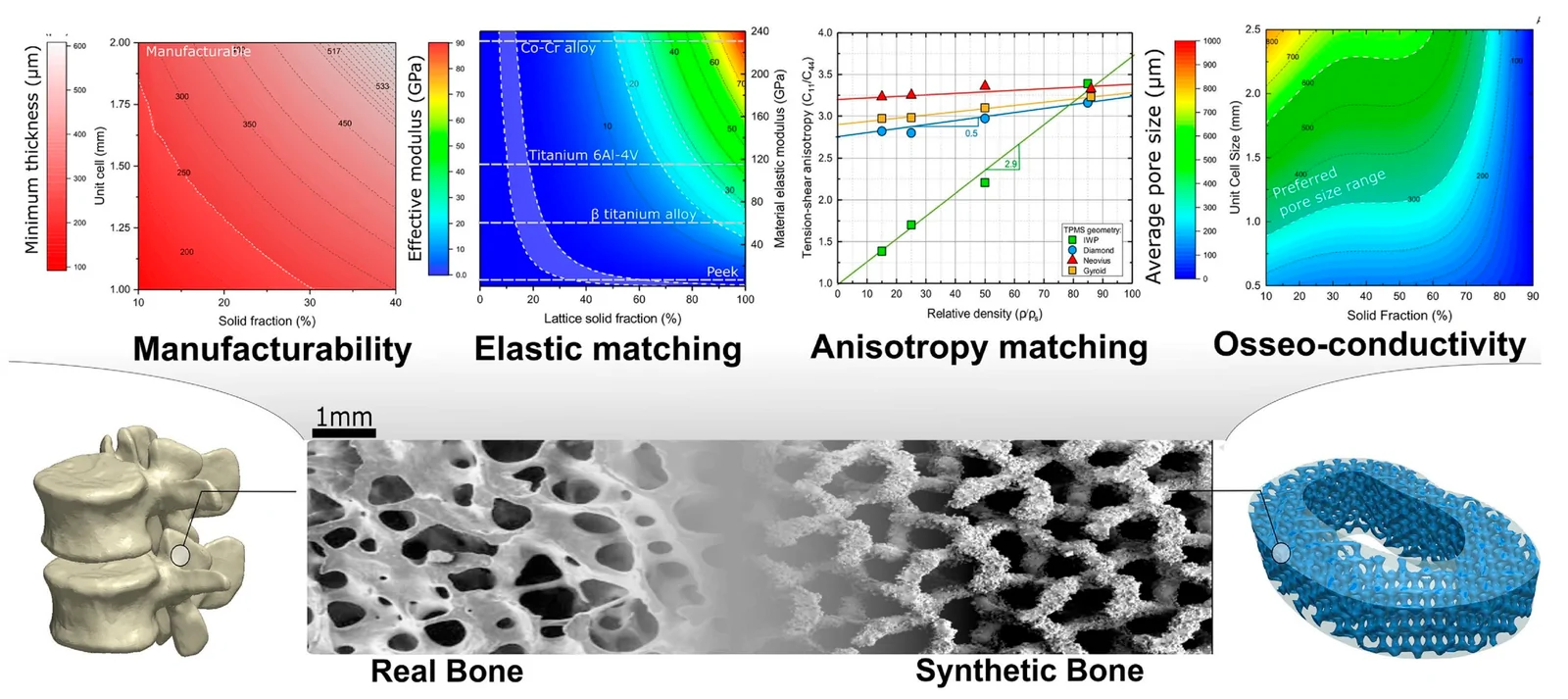
The combined properties of manufacturability, mechanical behavior, and osseointegration of the implant are matched to the properties of bone [28].

The manufacturability of the design is the premise of whether the bone implant can be successfully transformed. Because of the inherent limitations of additive manufacturing technology, the minimum feature size, powder removal and geometric accuracy must be fully considered in the design [27]. Barba et al. [28] found through micro-CT analysis that the minimum printable thickness should not be less than 250 μm in order to ensure acceptable geometric accuracy, and put forward the optimal design area based on manufacturability, mechanical properties and osseointegration, and its optimal design framework is shown in Figure 2. Prado et al. [29] compared the effects of additive manufacturing, traditional machining and acid etching on local bone mechanical properties after implantation in the human body. Experimental results showed that the average surface roughness of implants fabricated by additive manufacturing was significantly higher than that of traditional machining and acid etching treatment groups. This significant difference indicates that the additive manufacturing process tends to produce a surface with high roughness. Gaur et al. [30] systematically investigated the effects of laser power, scanning speed and scanning spacing on the mechanical properties of Ti-6Al-4V alloy formed by SLM through orthogonal experiments and a VIKOR multi-criteria decision-making method. The experimental results showed that the combination of moderate laser power, high scanning speed and low scanning spacing could make the yield strength, elongation and density meet the requirements of ASTM standards at the same time, which provided an experimental basis for the initial process parameter selection of customized bone implants for patients. The additive manufacturing technologies suitable for bone scaffolds were classified by Feng et al. into four categories—sheet stacking, wire stacking, particle stacking and powder stacking—and the influences of various technologies on structural design were analyzed one by one [31]. Table 1 summarizes the main characteristics of these four processes.

### 2.2. Key Structural Parameters

The structural design of bone implants has an important influence on their mechanical adaptation and the realization of biological function. Parameters such as pore size, porosity, gradient characteristics, anisotropy and surface characteristics jointly determine the interaction effect between implant and host bone tissue. Therefore, precise regulation of these parameters is the basis of designing high-performance bone implants.

**Figure 3:**
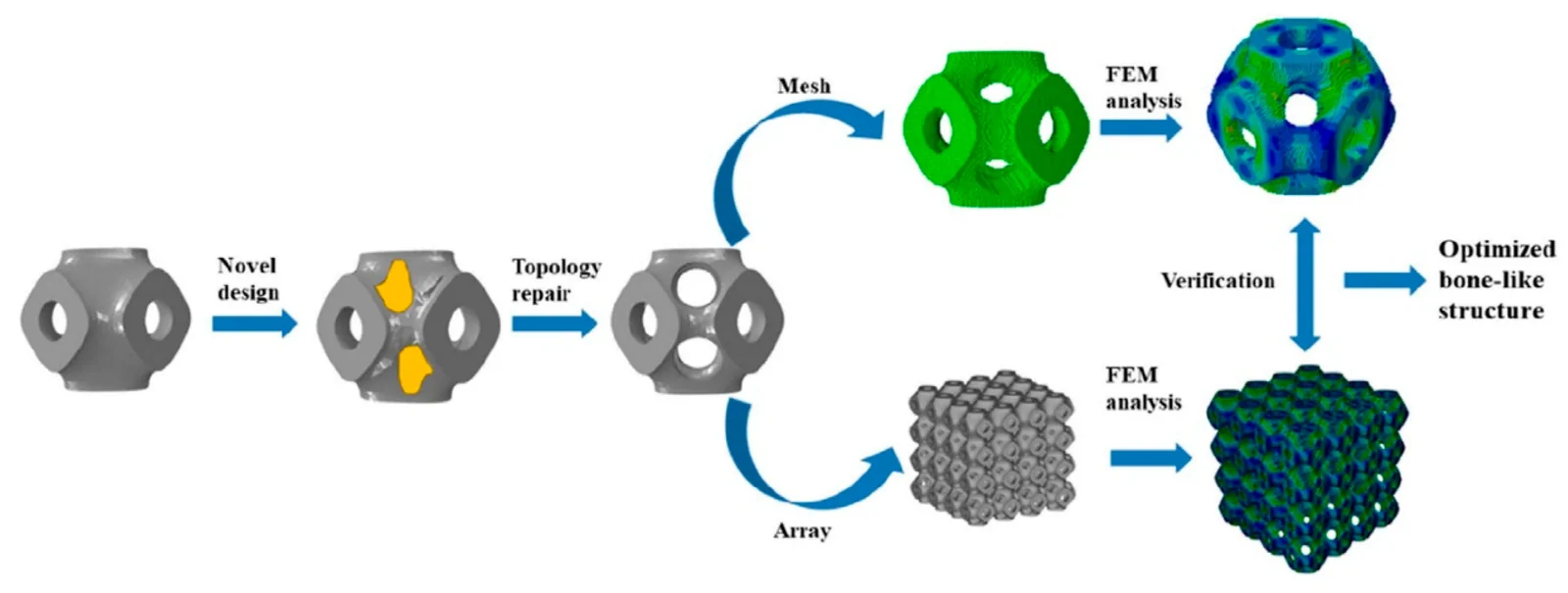
Schematic diagram of the design process of the novel structure based on the Schwarz P structure [33].

It is widely believed that porosity and pore size are the main factors affecting bone tissue growth, vascularization and nutrient transport according to a large number of in vitro and animal studies. Papazoglou et al. [32] investigated the effect of a Ti-6Al-4V lattice structure with different pore sizes based on additive manufacturing on the proliferation and differentiation of MG-63 osteoblasts through in vitro cell experiments. The results showed that the cubic lattice with 900 μm pore size was superior to other structures in glucose consumption, alkaline phosphatase activity and terminal cell count, and it also showed that larger pore size was more conducive to cell proliferation and material exchange under certain conditions. Based on the Schwarz P structure, Lai et al. [33] designed a new multifunctional gradient porous bone scaffold, and fabricated a new structure with different porosities through topology optimization and linear gradient transition. The design process of this new structure is shown in Figure 3. The results showed that the elastic modulus of the scaffold was more uniform in space and the elastic modulus difference in different directions was small. Compared with the traditional scaffold, it is closer to host bone, which is helpful to reduce stress shielding and is more suitable for the application of bearing parts.

Native tissue is not a homogeneous structure, but presents continuous porosity and mechanical property gradients from cortical bone to cancellous bone. Conventional homogeneous porous implants cannot simulate this natural gradient, leading to a sudden change in mechanical properties at the interface between implants and bone tissue, which will lead to stress concentration and interface failure. Functionally graded design has become the mainstream direction of bone implant design; it involves continuously changing the structural parameters of the implant in space to match its mechanical properties and biological functions with the gradient characteristics of the host bone. Gao Ruining and Li Xiang [34] designed a radial gradient porous scaffold based on a Gyroid surface, and realized the gradient distribution of mechanical properties by adjusting the porosity of the center and edge. FEM analysis and compression tests showed that the elastic modulus and compressive strength of the radial gradient scaffold were significantly better than those of the homogeneous porous scaffold under the same average porosity, and this advantage was more obvious when the average porosity was higher. Xu et al. [35] designed a bionic trabecular prosthesis with a radial gradient with the diameter of the hole edge for an infected bone defect in a tibia. Compared with the homogeneous structure, the finite element analysis results showed that the stress of the radial gradient structure prosthesis spread from the outside to the middle–high-porosity area along the prosthesis, which could effectively transfer the tibial plateau load and reduce the stress concentration of the prosthesis, thereby effectively relieving the stress shielding effect and prolonging the service life of the prosthesis.

Anisotropy and surface characteristics are important parameters for evaluating the mechanical properties and biological response of bone implants. Bai et al. [36] think that additive manufacturing technology can prepare implants with complex lattices, but different lattice topologies will show obviously different anisotropic mechanical behaviors. Regarding surface characteristics, this review summarized a variety of surface modification methods, which aimed to improve surface roughness and wettability and provide biological activity signals, thus promoting the adhesion, proliferation and differentiation of osteoblasts and improving the osseointegration ability of implants.

From the perspective of structural design methods, pore size and porosity are the most basic means of regulation, and the relationship between cell growth and mechanical bearing capacity should be balanced when regulating them. Too-small pore size restricts the inward growth of tissue, while too-large pore size will weaken the specific surface area and mechanical strength of the structure. With the introduction of gradient features, the implant can achieve differentiated performance distribution in space, but it also destroys the structural symmetry. Therefore, the anisotropy should be controlled according to the mechanical environment of the implant site. At present, research has accumulated substantial data on the influence of various parameters on performance, which has laid the foundation for the transformation from theoretical research to practical application in this field.

## 3. Structural Types and Design Methods of Bone Implants

Additive manufacturing technology can accurately construct complex geometric shapes, which has natural advantages in personalized customization and internal porous structural design of bone implants. The macro-shape and internal porous structure of implants are the keys to the long-term stability and functional recovery of implants. Therefore, research on the structural types and design methods of bone implants has always been a hotspot in the fields of biomanufacturing and bone tissue engineering.

### 3.1. Typical Structural Types of Bone Implants

The core advantage of additive manufacturing technology is that it can accurately and efficiently construct entities with complex geometric shapes, which provides a realistic basis for personalized customization of bone implants and refined design of internal porous structures. Research has shown that the macroscopic shape of the implant determines its initial matching degree with the host bone. The internal porous structure directly affects key biological processes such as mechanical properties, material exchange and new bone growth, and is the core factor in determining the long-term stability and functional recovery of implants [37]. Therefore, research on the structural types of bone implants has become a frontier hotspot in the fields of biomanufacturing and bone tissue engineering.

#### 3.1.1. Regular Porous Structure

Regular porous structures are usually formed by periodic arrangement of basic units such as cubes, hexahedrons and rhombic dodecahedrons in three-dimensional space. They are widely studied because of their simple structure and convenient theoretical analysis and finite element simulation. The mechanical and physical properties of these kinds of structures are mainly determined by the element geometry, aperture and rod diameter, and they are highly repeatable and predictable.

**Figure 4:**
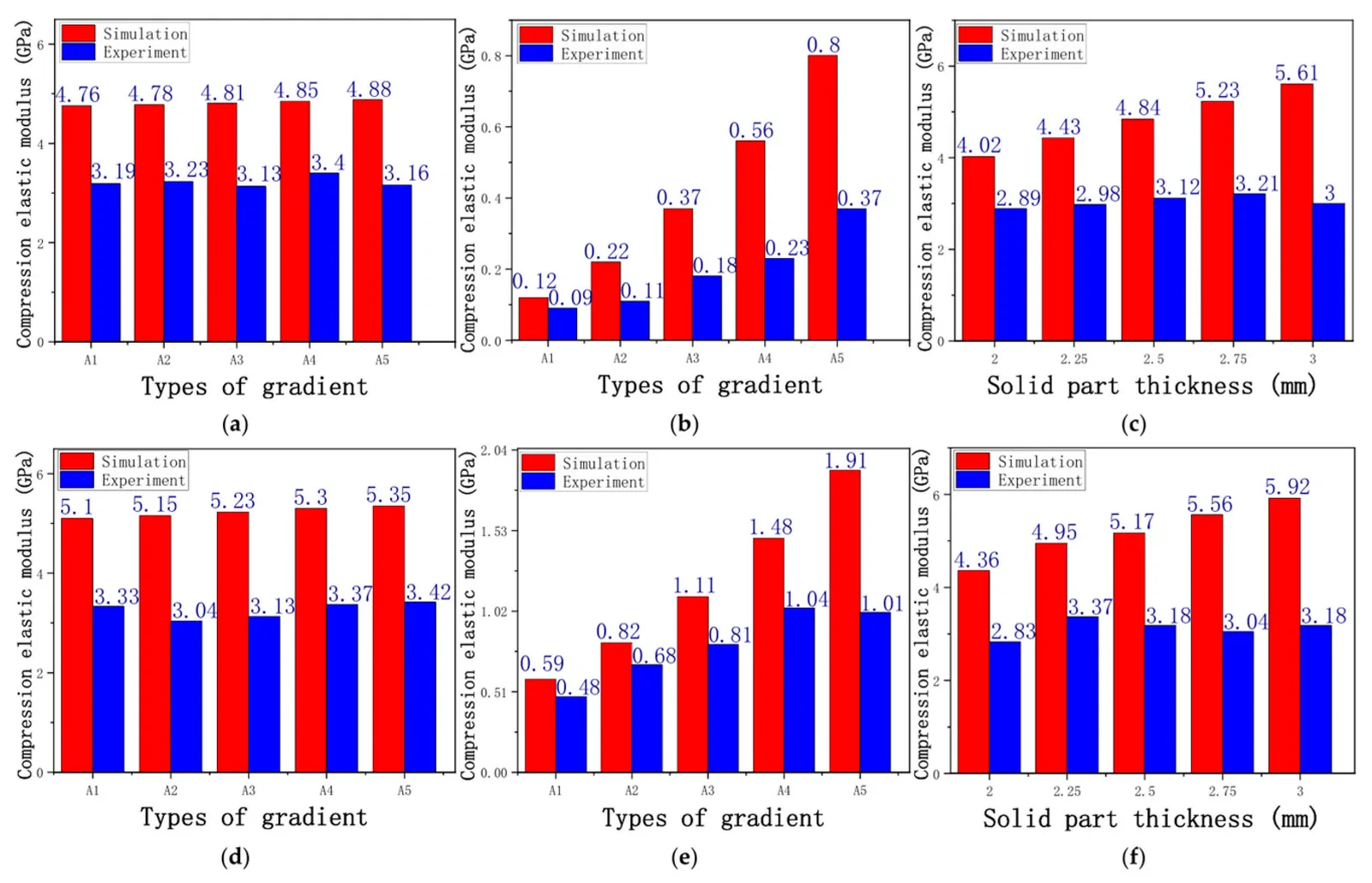
Bar chart of actual elastic modulus and simulated elastic modulus of rhombic dodecahedron and derived dodecahedron. (**a**) Rhombic dodecahedron perpendicular to the direction of structural gradient compression. (**b**) Rhombic dodecahedron parallel to the direction of structural gradient compression. (**c**) Rhombic dodecahedron solid part thickness variation. (**d**) Derived dodecahedral perpendicular to the direction of structural gradient. (**e**) Derived dodecahedral parallel to the direction of structural gradient. (**f**) Derived dodecahedron solid part thickness variation [39].

Through quasi-static compression experiments, Zhong et al. [38] studied the mechanical behavior of a titanium alloy Ti-6Al-4V bone scaffold with regular hexahedral units and a layered gradient structure, and found that the porosity of the uniform structure could change from 40.91% to 84.89% by adjusting the rod diameter to 0.3–0.7 mm. Therefore, its equivalent elastic modulus can be adjusted to the range of 1.81–14.12 GPa, which can effectively match the mechanical properties of human humerus. In addition, compared with the uniform structure, the gradient structure shows more uniform stress distribution under shear load, and can control the direction of stress transmission more effectively, thus avoiding the risk of failure caused by stress concentration. This conclusion is based on standard sample tests under uniaxial loads, and its actual matching effect under complex physiological loads still needs further in vivo and clinical verification. Zhai et al. [39] compared uniform and gradient models of a rhombic dodecahedron and its derivative structure, and found that the porosity of the derivative structure was more sensitive to a change in rod diameter. The elastic modulus was better than that of the rhombic dodecahedron under compression and shear conditions. Figure 4 is a bar chart comparing the simulated elastic modulus value with the actual elastic modulus value. Limmahakhun et al. [40] further enriched this field. They compared the performance of an octahedron, columnar octahedron, cube and truncated octahedron under compression, shear and torsion loads, and found that the columnar octahedron structure with vertical pillars had the highest comprehensive stiffness and strength. Simultaneously, it promoted a higher osteoblast proliferation rate in vitro, showing a better balance between mechanics and biology.

The advantage of a regular porous structure lies in its strong designability, and its mechanical properties can be adjusted by adjusting the type and size of units. However, due to the difference between the highly uniform geometric characteristics of this structure and the irregularity of native tissue, it has brought some limitations to its long-term adaptability in complex physiological environment, but this has also spawned new structural designs with more bionic characteristics.

#### 3.1.2. TPMS Structure

TPMS is a kind of mathematical surface with periodicity in three independent directions and zero mean curvature. Compared with a regular porous structure, the TPMS structure is completely composed of a continuous curved surface without sharp corners, which not only has an extremely high specific surface area and completely connected pores, but also shows excellent permeability and bionic potential. In recent years, it has become a research hotspot in the field of bone implant design.

Han et al. [41] found through computational fluid dynamics (CFD) simulation that the permeability of the TPMS structure was about 20 times higher than that of the traditional rod-shaped truss structure, which significantly improved the efficiency of nutrient transport and metabolic waste discharge. This high permeability is mainly due to its smooth and continuous surface, which can reduce resistance to fluid. The review by Ma et al. [42] also systematically summarized the comprehensive influence of microstructure parameters such as geometry, wall thickness, pore size, porosity and surface curvature of TPMS on bone regeneration, and emphasized the superiority of radial gradient TPMS scaffolds in simulating native structures. In addition, the mechanical properties of the TPMS structure are highly controllable and can match native tissue. The porous titanium alloy scaffold based on a Gyroid solid network structure was used to repair metacarpal defects by Liu et al. [43], and the Gyroid structure with a target porosity of 70% was combined with the patient defect area model through Boolean operation and personalized preparation. The results showed that the elastic modulus of the scaffold was similar to that of human cancellous bone, and cell experiments in vitro also confirmed that it could significantly promote the expression of ALP and OPN-related genes in late osteogenesis. Tsai and Chang [44] used a particle method based on Lennard–Jones potential to simulate the process of screw insertion and extraction in TPMS bone implants. It was found that the ratio of pullout strength to maximum insertion force of implants with a Gyroid structure and Fisher–Koch S structure was much higher than that of solid structures. It showed that the TPMS structure could provide better screw fixation stability by limiting the damaged area. The various types of TPMS structures also provide the possibility for personalized customization of bone implants. Karamanli [45] optimized a functionally graded hybrid structure composed of the inner and outer layers of different TPMS structures by using the design-of-experiments method and Taguchi method. The results showed that the mixed combination of Neovius and Diamond had the best bearing capacity and energy absorption, and the type and filling density of the external structure had the most obvious influence on the mechanical properties.

The above research shows that the performance of the TPMS structure can be accurately regulated by its implicit function equation. This makes this kind of structure show obvious advantages in mechanical bionics and biological functions, and becomes an important bridge connecting regular porous structures with complex bionic structures.

#### 3.1.3. Gradient and Composite Structure

Inspired by the gradual structure of native tissue from dense cortical bone to porous cancellous bone, researchers began to study gradient and composite structures. This structure can eliminate the sudden change in mechanical properties of the uniform structure at the interface and realize a smooth transition between the implant and the host bone. In order to simulate the characteristics of this structure, researchers have designed a variety of gradient porous structures, including configurations in which porosity, pore size or unit type changes continuously or stepwise in a certain direction. Tüzemen et al. [46] established a mathematical model to predict the porosity and elastic modulus of a stepped radial gradient structure, and found that the stress transfer path in the gradient structure was more concentrated and controllable than that in the uniform structure, which was beneficial for reducing stress shielding. Zhang et al. [47] systematically studied the influence of material composition, namely a PEEK/hydroxyapatite (HA) composite, structural design and manufacturing process on the permeability and mechanical properties of the scaffold. The results showed that the permeability and mechanical properties of PEEK/HA scaffolds with a relative density of 35% to 50% were similar to those of human cancellous bone, which provided the design possibility for balancing mechanical and biological properties.

Composite structures often combine solid parts and porous parts, or integrate units with different topological structures. Mohammadi et al. [48] proposed a gradient structural design and performed optimization research based on auxetic cells to solve the stress shielding problem of hip implants. By using the characteristics of lateral expansion of the tensile expansion structure to increase the contact area between the implant and the bone, the elastic modulus increased from the bone contact surface to the interior of the implants, thus ensuring the high strength of the internal core area. Gao et al. [49] used a Kriging model to establish the mapping relationship between the adjustable size of a porous structure and porosity, specific surface area and elastic modulus, and optimized the equivalent stress of a femoral surface by the response surface method. Finally, the gradient porous femoral stem was designed, which increased the strain on the femoral surface by 17.1% on average and effectively relieved the stress shielding effect.

#### 3.1.4. Bionic Natural Structures

**Figure 5:**
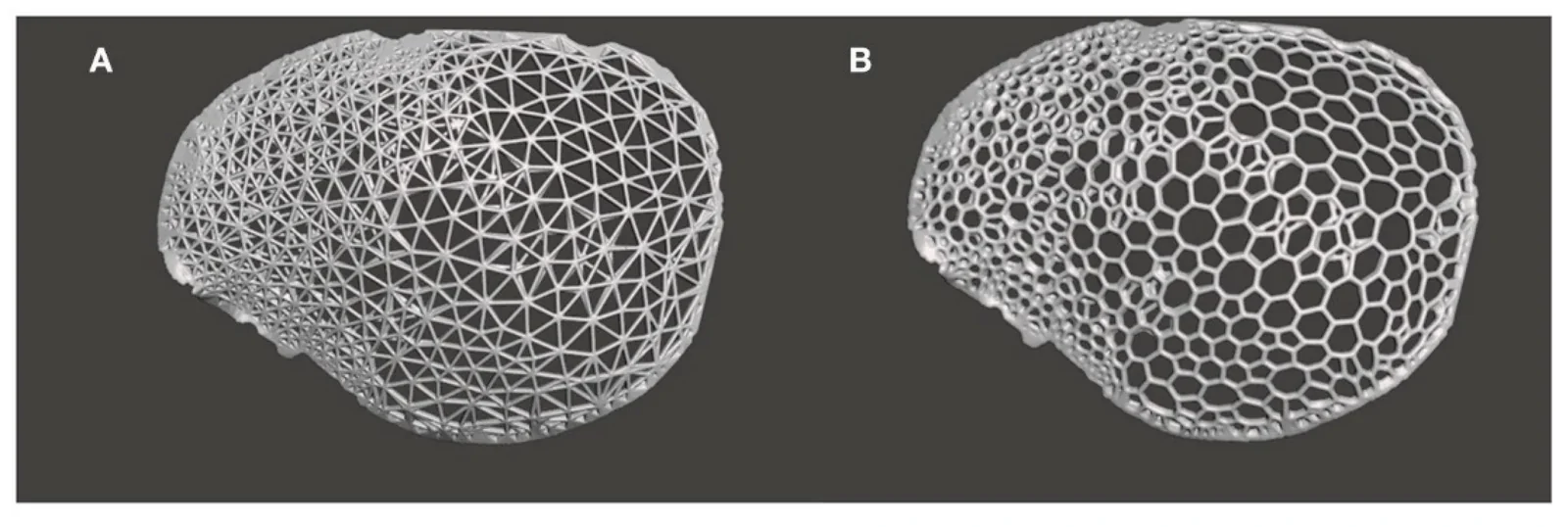
(**A**) Wireframe/lattice Voronoi prosthesis design generated using the “Mesh + Delaunay Edges” algorithm. (**B**) Organic Voronoi prosthesis design developed using the “Mesh + Delaunay Dual Edges” algorithm [51].

Bionic natural structures are the highest level of bionic design; they can fully replicate the characteristics of native tissue from macro-shape to micro-configuration. Their design inspiration comes from the efficient structure and function of organisms. After finely quantifying the longitudinal and radial microstructure gradient of a rabbit femur by high-resolution computerized tomography, Zhao et al. [50] found that the porosity gradually increased from the outer surface of the bone to the inside, and also showed an increasing trend from the middle to both ends along the femur. This study provides an important structural benchmark and data support for designing bionic implants with a stiffness gradient completely matching that of native bones. Bionic structures based on Voronoi diagrams have attracted many researchers’ attention because they can simulate the irregularity and anisotropy of cancellous bone. Sharma et al. [51] applied this method to design personalized titanium alloy implants for skull repair. They used two algorithms of “Mesh + Delaunay Edges” and “Mesh + Delaunay Dual Edges” to generate two different Voronoi structures, and their modeling is shown in Figure 5. It was proved by selective laser melting technology that the latter organic Voronoi structure has better self-support and printing quality than the simple Voronoi structure. Pei et al. [52] introduced a quasi-homogeneous heterogeneous structure on this basis, and introduced controllable heterogeneity by applying random displacement to nodes on a regular Diamond structure. The research showed that 50% heterogeneity could not only keep the good overall mechanical properties of regular porous structure, but also simulate the micro-heterogeneity of native bones through local pore size and curvature fluctuation. In addition, in vitro and in vivo experiments have confirmed that this semi-isomeric structure was superior to completely uniform and completely random structures in promoting cell proliferation and new bone formation. The research of Li et al. [53] also embodies the idea of bionics, and they have prepared bionic gradient scaffolds and reverse gradient scaffolds from outside to inside. The results showed that the bionic gradient scaffold promoted higher alkaline phosphatase activity and osteogenic gene expression in vitro. In vivo experiments also confirmed that it could promote the new bone formation more effectivel.

Structural design of bone implants has developed from simple regular porous structures to accurately controllable TPMS structures and highly bionic irregular structures. There are significant differences among the four types of porous structures in mechanical properties, processing feasibility and clinical application scenarios. A single structure cannot meet all the requirements of low stress shielding, high fatigue life, rapid bone growth and low-cost mass production at the same time. To clearly illustrate the advantages and disadvantages of different structures as well as the existing research discrepancies, this paper provides a multidimensional comparative analysis of the four types of porous structures used in additive manufacturing for bone implants, as shown in Table 2.

### 3.2. Design Method of Bone Implant Structure

Structural design of bone implants is an important link for realizing its clinical application. With the increasing ability of additive manufacturing technology to manufacture complex geometric structures, the structural design of bone implants has been developing from the traditional standardized solid structural framework to the personalized, porous and functional direction. In clinical practice, achieving optimal implant design requires an integrated and multi-stage workflow, rather than a single solution. Its core feature lies in the complementary and iterative relationship of three methods, namely anatomical adaptive design based on CT/MRI image reconstruction, topology optimization design and additive manufacturing-oriented design. Image reconstruction methods provide the anatomical basis and boundary conditions necessary for performance optimization; topological optimization enables the optimal distribution of materials with regard to mechanical requirements; designs tailored for additive manufacturing offer a pathway for structural optimization. The three factors advance step by step and interact with each other to jointly support the research and development of high-performance personalized bone implants. The following will systematically expound on the design methods, research progress and collaborative mechanisms in this field from these three aspects.

#### 3.2.1. Anatomical Adaptation Design Based on CT/MRI Image Reconstruction

To achieve a complete anatomical match between bone implant and host site, 3D reconstruction technology based on CT and magnetic resonance imaging (MRI) images has become the core foundation of current design methods by simulating the mechanical environment of native bone. This method provides a data source and design basis for fabricating personalized and highly adaptable bone implants by extracting the anatomical data of patients.

**Figure 6:**
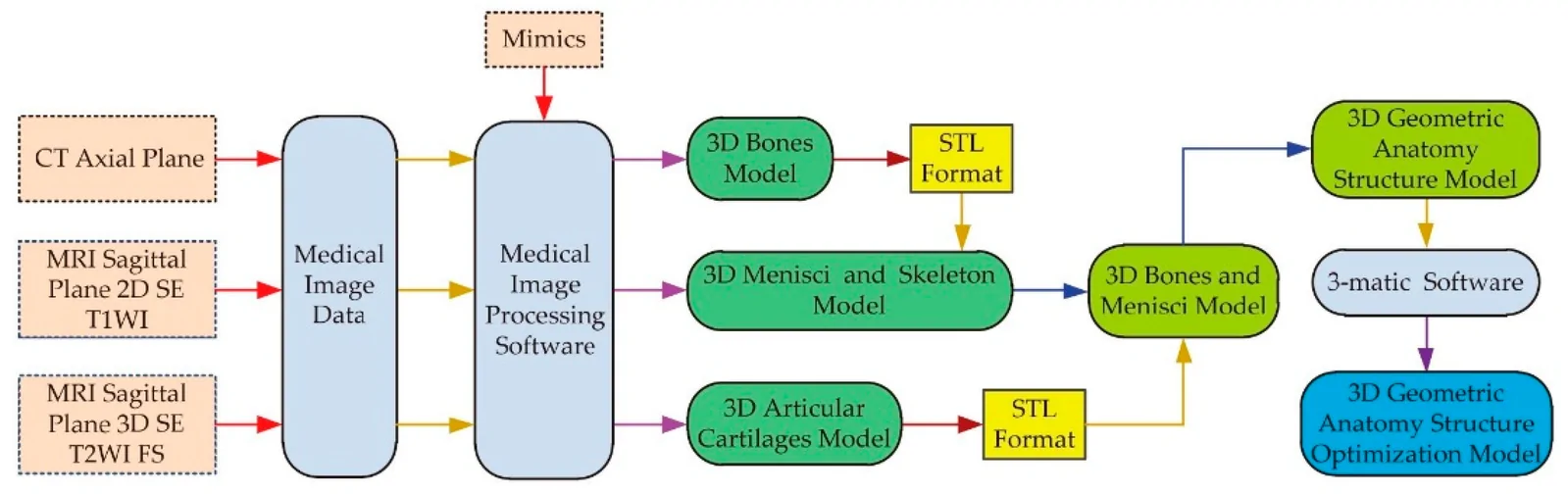
The framework of a 3D modeling methodology based on CT and two sets of different MRI sequences images [54].

In anatomical structure modeling, the application of multi-modal image data fusion is the key strategy to improve the fidelity of the model. For joints with complex structures, such as human knee joints, it is difficult to accurately present bone and soft tissue simultaneously with single-modality image data. Niu et al. [54] proposed a 3D modeling method based on CT and two different MRI images, and its 3D modeling framework is shown in Figure 6. Using high-resolution CT images, they reconstructed bony structures, such as the distal femur and the proximal tibia, and combined 3D models from different sources by manual and automatic global registration according to the specific imaging advantages of different MRI sequences for soft tissues. Finally, a high-precision geometric anatomical model of the knee joint including bone, meniscus and articular cartilage was fabricated. The method effectively integrates the advantages of CT and MRI, and provides an accurate anatomical framework for designing bionic bone implants. Rana et al. [55,56] systematically expounded a patient-specific scaffold design method based on CT image Hounsfield Units (HU). The method first converted the HU value of each pixel in CT data into the elastic modulus and density of bone tissue by an empirical formula, so as to realize the position-specific mapping of material properties in the finite element model. Subsequently, the simulated bone defect area was removed from different parts of the femur, so that the stiffness of each position of the porous implant was equal to that of the removed primary bone. The image-based design method realizes the dual bionics of shape and stiffness, and its clinical feasibility and effectiveness have been verified in practical cases. Yong et al. [57] applied 3D-printed patient-specific prostheses to bone defect reconstruction after resection of ankle tumors. In their design process, firstly, the CT and MRI data of patients were processed and 3D models were built, and then a patient-specific osteotomy guide plate was designed to guide accurate osteotomy. For three different types of cases—neurofibroma of the calcaneus, low-grade chondrosarcoma of the distal tibia, and fibular metastatic tumor—studies have shown that personalized prostheses designed by preoperative image data can achieve accurate anatomical adaptation and position fixation during surgery.

Anatomical adaptation design based on CT/MRI images can realize the dual bionics of the implant and host bone in macro-morphology and overall stiffness by transforming medical image data into personalized geometric and material distribution models. Its advantage lies in its “patient-centered” precise medical concept, and it can be customized for individual anatomical differences. However, its limitations are obvious: On the one hand, this method mainly relies on stiffness matching under linear static assumption, and fails to fully consider the nonlinear, viscoelastic and anisotropic characteristics of bone tissue. On the other hand, its control ability at the microstructure level is limited, and it struggles to achieve fine control of local mechanical conduction paths and the biological fluid environment through simple changes in the diameter of pillars. Thus, this method provides a good foundation of geometry and initial distribution of materials for further advanced optimization design, but it needs to be combined with more forward-looking structural optimization technology to further improve the comprehensive performance of implants. In collaborative application with the other two design methods, the advantage of design based on CT/MRI is that it can generate patient-specific anatomical models and provide preliminary stiffness distribution based on actual anatomical data. However, its main limitation is that it is impossible to carry out real structural optimization for complex load cases. The structure type of the topology optimization output and the minimum feature constraint of additive manufacturing can guide the simplification and smoothing of image reconstruction models, and avoid the limitation that too-fine anatomical details cannot be manufactured or cause stress concentration. Therefore, anatomical reconstruction is not an independent solution, but a necessary basic input for more advanced structural optimization.

#### 3.2.2. Topology Optimization Design

Among the structural design methods of bone implants, topology optimization, as a technology for achieving the optimal material distribution in a given design domain through mathematical algorithms, has been widely considered in recent years. This method is driven by mechanical load and boundary conditions, and minimizes structural compliance under the premise of satisfying volume or stress constraints, so as to obtain a lightweight structure with excellent mechanical properties. As for bone implants, topology optimization can not only reduce the weight of implants and alleviate stress shielding, but also combine with porous structure and bone remodeling theory to make up for the shortcomings of the previous methods in micromechanical optimization.

**Figure 7:**
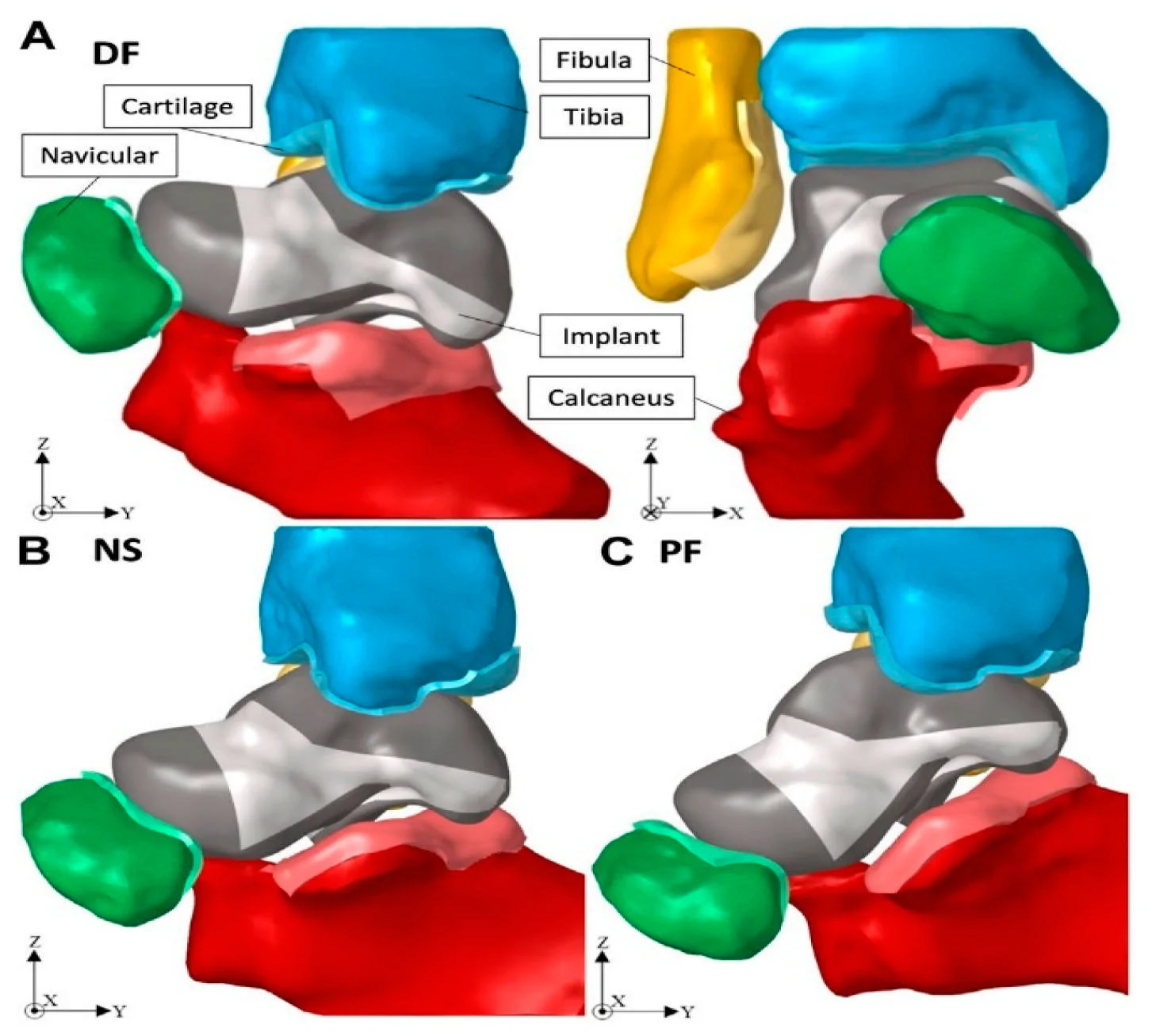
Ankle joint in (**A**) +20° DF, (**B**) 0° NS, and (**C**) −20° PF [62].

Zhu et al. [58] proposed a 3D-printed prosthesis design method combining topology optimization and porous structures to meet the reconstruction requirements after resection of bone tumors around the acetabulum. They established a finite element model based on the CT data of patients, and used a solid isotropic material penalty model to optimize the topology of the prosthesis. Research results showed that after topology optimization, the strain energy densities of type I and type II prostheses were significantly increased by 143.61% and 35.05% respectively, indicating that the optimized prostheses could transmit mechanical stimulation more effectively, thus reducing the risk of stress shielding. In the treatment of proximal femoral bone defects, Xue et al. [59] designed a composite sleeve and intramedullary pin handle prosthesis, and used density-based topology optimization to redistribute the material of the sleeve part. FEM analysis showed that the maximum equivalent stress of the femur in the optimized group was increased by 29.52% compared with the original group, while the maximum stresses of the posterior sleeve and the stem and neck of the prosthesis were decreased by 30.33% and 4.74% respectively, which were beneficial to bone growth. It is proved that the strategy of combining topology optimization with a gradient porous structure can improve stress transfer and promote osseointegration on the premise of ensuring mechanical strength. In mandibular reconstruction, Peng et al. [60] proposed a layered porous titanium implant and designed a fixed plate structure by the topological optimization method. Firstly, the mandibular defect model was reconstructed based on CT images, and then a porous implant with a three-layer structure of “Layered Slice and Rod Connected Mesh Structure” was fabricated. Finally, the force transmission path was determined through two topological optimization iterations, and finally a configuration of a wing plate and force transmission pillar was formed. This study extends the application of topology optimization from implant body to its fixed system, which reflects the global optimization ability of this method in complex structural systems. In order to predict the process of bone remodeling, Mathai et al. [61] established a computational framework based on multi-scale topology optimization and a parameterized cell model to simulate the changes in bone adaptability around proximal femoral implants. The microstructure model of “Shi Ying Crystal” was adopted in this study, and the orientation of orthotropic materials was determined by the direction of principal stress. The topological optimization results were compared with the traditional isotropic strain energy density model and anisotropic remodeling model based on principal strain. Regression analysis showed that the three models predicted different degrees of bone resorption in the proximal femur, but the topological optimization model predicted higher bone hyperplasia in the distal femur, indicating that this method can capture the anisotropic remodeling behavior of bone. In the field of talus total replacement, Hafez et al. [62] proposed a lightweight general talus implant design method based on topology optimization. This study established the finite element models of three foot postures—neutral position (0°), dorsiflexion (+20°) and plantarflexion (−20°)—and considered the load conditions in these three postures. The finite element settings of ankle joints in three foot postures are shown in Figure 7. Compared with previous models based solely on simplified loading conditions, this model exhibits significantly improved anatomical fidelity. In terms of mechanical performance verification, the maximum von Mises stress of the optimized implant in all three postures did not exceed 70 MPa, and the corresponding minimum safety factor (in flexion posture) was 15.7, which was much higher than the design requirements of conventional engineering structures.

The above research shows that the application of topology optimization in bone implant design is developing from single-structure weight reduction to multi-functional integration. Its core advantage is that it can automatically generate non-intuitive and efficient material distribution under given design space, load and constraint conditions, so as to achieve the optimal balance between mechanical properties and biological properties. In particular, combined with porous structures such as TPMS and lattices, topology optimization can generate complex configurations with gradient porosity. In addition, topology optimization is coupled with bone remodeling theory and a parameterized microscopic model, which provides a new calculation tool for predicting and guiding the changes in bone adaptability after implantation. However, there are still some limitations in the existing research: most optimizations are based on static linear loading conditions, ignoring the influence of dynamic and multi-axial fatigue loads in daily activities. What is more, the idealized geometric configuration generated by topology optimization often contains details such as overhanging structure, minimal size features and so on, which are difficult to realize directly by additive manufacturing. Therefore, how to internalize process constraints into penalty functions or constraints in the optimization model is the core challenge facing the current methods. Topology optimization is the core function of mechanical performance optimization in the structural design of bone implants. The anatomical model and load environment constructed by CT/MRI images must be reconstructed to ensure that the optimization results match the individual physiological environment of patients. The optimal material distribution and force transmission path obtained by topology optimization also provide clear structural guidance for the subsequent design for additive manufacturing, and avoid the blindness of structural design. Meanwhile, in order to improve the manufacturability of the optimization results, the key process constraints such as minimum wall thickness, hanging angle and supportability of additive manufacturing are gradually becoming important factors in the topology optimization algorithm.

#### 3.2.3. Design Based on Additive Manufacturing

Traditional manufacturing methods are difficult to accurately control the shape, size and spatial distribution of pores, while additive manufacturing allows complex computer-aided design (CAD) models to be directly transformed into physical entities. However, the design for additive manufacturing not only needs to pursue the functional optimization of the structure like topology optimization, but also must fully consider the influence of technological process on molding accuracy, material properties and structural integrity. Therefore, the manufacturability constraint is integrated in the design stage, and the theoretically optimal structure is turned into a printable and applicable implant in reality.

Zhou et al. [63] systematically reviewed various additive manufacturing technologies and their effects on structural design in the field of bioceramic implants. Among them, photopolymerization technology had the highest printing accuracy and was suitable for preparing fine ceramic porous structures. Direct ink writing (DIW) technology had low cost and wide application range of materials, but its molding accuracy was poor, and it was difficult to print fine structures, and the extruded structure was highly limited by mechanical stability. Fused Deposition Modeling (FDM) technology was limited to thermoplastic polymer or ceramic composites. These technical characteristics require designers to compensate for the accuracy limit, material requirements and post-treatment shrinkage of the selected process when designing the structure of bone implants. In the aspect of the influence of additive manufacturing process on structural molding, Seidenstuecker et al. [64] compared β-tricalcium phosphate (β-TCP) bone implants prepared by traditional sintering and additive manufacturing methods. They found that although the porosity of the hybrid structure made by additive manufacturing was as high as 74.4%, the compressive strength was close to that of human cancellous bone, while the traditional microporous implant had higher strength but lower porosity. Roque et al. [65] discussed the decisive role of printing parameters on molding quality. Two scaffold models with different pore sizes and porosities were designed. It was found that the optimization of printing parameters was the key to obtaining good interlayer adhesion and structural integrity. The research clearly points out that the design for additive manufacturing must respect the resolution limit of equipment and the rheological characteristics of materials, and the complexity of design needs to achieve a realistic balance with manufacturing capacity. Laser Powder Bed Fusion (L-PBF) technology has become the first choice for manufacturing high-performance porous titanium alloy structures for metal implants. Kelly et al. [66] prepared Gyroid titanium alloy implants with different porosities by LPBF technology, and systematically evaluated their mechanical properties and osseointegration effect in sheep. The research showed that the compressive modulus and strength decreased linearly with the increase in porosity, covering the mechanical range from cancellous bone to cortical bone. In addition, the surface roughness and partially adhered spherical powder particles produced by the LPBF process formed a three-dimensional morphology conducive to the adhesion of bone cells. It shows that the inherent geometric deviation of additive manufacturing needs to be compensated in design. In the aspect of multi-morphological gradient structural design, Yu et al. [67] compared the mechanical and permeability properties of four TPMS structures through finite element analysis, and found that the maximum von-Mises stress of the Primitive structure was the lowest, while the permeability of the Gyroid structure was the highest. Both of them were complementary in mechanical properties and mass transfer properties, and the gradient composite structure with better performance was obtained by combining them using the selective laser melting process. These design methods require designers to have cross-domain knowledge and be able to transform process factors such as printing accuracy, material shrinkage, supporting structure and powder removal into geometric constraints at the front end. Meanwhile, designers make full use of the topological freedom and surface morphology control ability brought by additive manufacturing in the design.

The design for additive manufacturing is at the end of the whole design process. Its main function is to carry out process-oriented geometric correction and manufacturability verification based on the anatomical model of the image reconstruction output and the excellent structure of the topology optimization output. The feedback of the process limitation can be transmitted to the first two links in reverse: on the one hand, it guides the image reconstruction to simplify the tiny anatomical features that are difficult to shape; on the other hand, it drives the topology optimization to adjust the constraints and regenerate the configuration that meets the process requirements. Based on the structural design ideas of the three types of implants in this section and combined with Table 3, it can be seen that each method has its own strengths in terms of functionality, and none of them can meet all the design requirements on their own. Image reconstruction technology can better adapt to the shape of human bones, but its mechanical control ability is insufficient. Topology optimization can improve the stress distribution, but the structure generated by this method is prone to collapse, size deviation and other problems. The additive manufacturing process can only correct the molding defects, but it cannot adapt to the patient’s personalized anatomical modeling and load requirements. The combination of the three can balance design, mechanical adaptation and machining feasibility.

## 4. Effect of Structural Design on Key Performance of Implant

The structural design of bone implants is directly related to the mechanical adaptation and biological response of implants in vivo. On the one hand, the implants need to match the elastic modulus of the host bone to avoid bone resorption caused by stress shielding. On the other hand, its internal pore structure must provide a suitable microenvironment for cell adhesion, proliferation, differentiation and tissue growth. However, a too-sparse pore structure is beneficial to bone growth, but it may lose mechanical stability; a too-dense structure can ensure strength, but it may hinder tissue regeneration. Thus, exploring how structural design affects the key performance of implants has become the focus of the structural design of porous bone implants.

### 4.1. Adjustment and Control of Mechanical Properties by Structural Design

#### 4.1.1. Elastic Modulus Matching and Stress Shielding Relief

In the clinical application of bone implants, the stress shielding effect caused by the mismatch of the elastic modulus between implant and host bone is the core problem in the structural design of bone implants. The stiffness of traditional dense metal implants is much higher than that of human native bones, which makes the implants bear most of the mechanical loads, leading to the gradual shrinkage of surrounding bone tissues due to the lack of necessary stress stimulation. In order to solve this problem, it has become a hot research topic to adjust the macro elastic modulus of implants through structural design to match native bones.

Porosity is widely regarded as the key parameter affecting the elastic modulus of porous structures in calculation and experimental models. In a finite element parametric study of a Ti-6Al-4V Voronoi structure, Chao et al. [68] found through systematic parametric research that when the porosity of the Ti-6Al-4V Voronoi porous structure increased from 50% to 90%, its elastic modulus could be reduced from 7.42 GPa to 0.49 GPa, which completely covered the modulus range of host bones. Ganesh and Rambabu [69] used finite element analysis to systematically study the influence of different porosities on the elastic modulus of Ti-6Al-4V porous cylindrical samples. The results showed that when the porosity increased from 10% to 40%, the elastic modulus gradually decreased from 97 GPa to 70 GPa. The above research proves that the stiffness of implants can be customized within a certain range by adjusting porosity, thus effectively relieving stress shielding. However, the actual effect of relieving stress shielding in vivo still needs to be further verified by mechanical experiments and clinical experiments.

**Figure 8:**
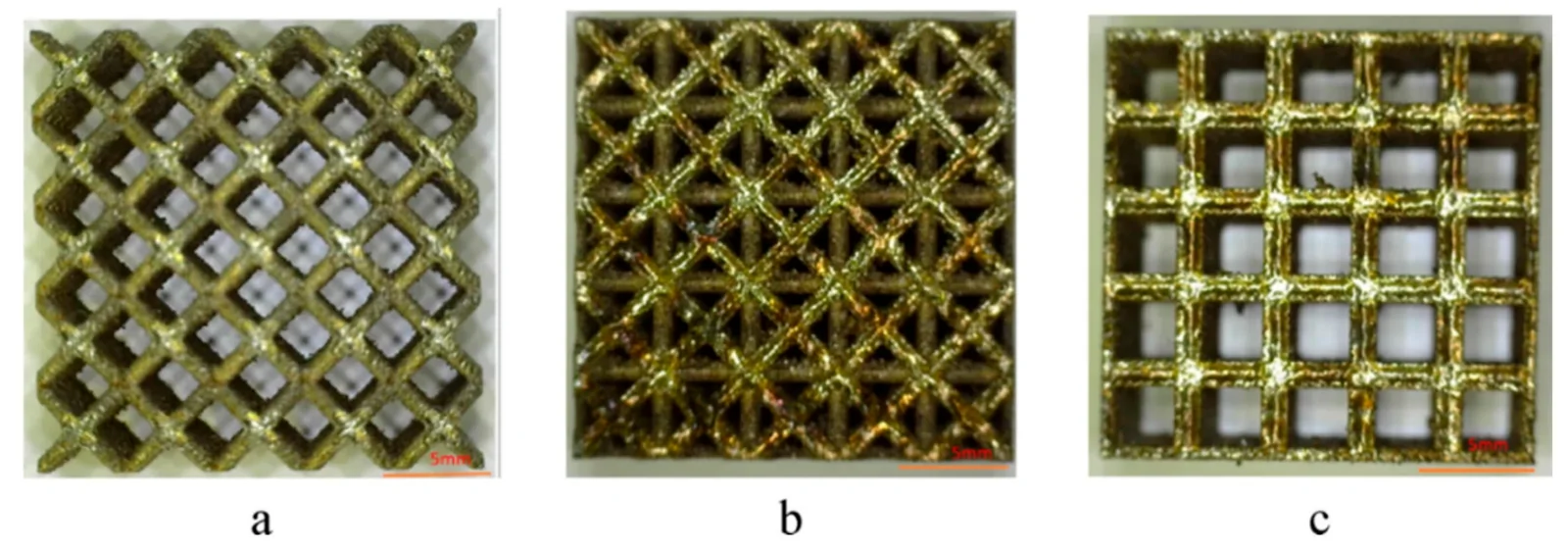
Top view of the printed specimens: (**a**) BCC, (**b**) FCC, (**c**) SC [70].

Rajabi et al. [70] focused on the lattice structure of stainless steel 316 L. Through finite element simulation and quasi-static compression experiments, three kinds of lattice structures of stainless steel 316 L were compared under the same nominal relative density: BCC, FCC and SC. It was found that the elastic modulus of the SC structure was 1.3 times and 4 times that of FCC and BCC, respectively. Figure 8 shows the printed samples of three structures. The reason for this difference was that the load of the SC structure was mainly transmitted through the tension/compression of struts, while the BCC and FCC structures contained more inclined struts, which were prone to bending deformation during compression. It was shown that the SC structure not only provided the closest elastic matching to cancellous bone, but also had the highest compressive strength, which provided a feasible topological scheme for the design of bone implants with low stress shielding and sufficient bearing capacity. Sunavala-Dossabhooy et al. [71] improved the load distribution by trussed “material reduction” design inside the femoral shaft, and increased the von Mises stress ratio of the proximal medial bone tissue from 26% to about 36% of the solid shaft. The relative density gradient matched with the local stress field was generated by Wang [72] using proportional topology optimization. It was found that the predicted bone resorption of gradient lattice implants was only 78.8% of that of uniform lattice implants, which was 58.1% less than that of solid implants. The advantage of this strategy is that the high-density area is arranged in the main bearing path to ensure the fatigue strength. However, the low-density area is located in the high-risk area of stress shielding, such as the proximal inner side, which forces more stress to be transmitted to the surrounding bone tissue.

It can be seen that reducing porosity or relative density is the most direct means to reduce modulus by adjusting the elastic modulus through structural design to relieve stress shielding, while changing cell topology can obtain higher stiffness at the same density. Meanwhile, associating porosity or structural type with local stress distribution has been proven to be a better strategy than a uniform porous structure, which can ensure the mechanical stability and promote the biomechanical adaptation between implant and native bones to the maximum extent.

#### 4.1.2. Regulation of Porosity and Pore Size on Elastic Modulus

In the structural design of bone implants, porosity and pore size are the core parameters that determine their mechanical compatibility, especially their elastic modulus. Ideal bone implants need to have an elastic modulus similar to that of the host bone to avoid bone resorption and implant loosening caused by the “stress shielding” effect.

H. A. Zaharin et al. [73] found that the elastic modulus of Ti-6Al-4V decreased with the increase in porosity by comparing the cubic structure with the Gyroid structure. The porous sample of 316 L stainless steel prepared by Khamis Essa et al. [74] by hot isostatic pressing without jacketing increased the porosity control from 1.4% to 25.4% by changing the initial powder diameter, and its elastic modulus correspondingly decreased from 50 GPa to 17 GPa. It is shown that the modulus range of bone matching can be obtained only by adjusting the particle stacking state, even without relying on complex lattice design. In the lattice structure of radial-gradient TiNi designed by Tan et al. [75], the elastic modulus changed continuously between 1.65 and 4.46 GPa, which just covered the modulus range from cancellous bone to cortical bone. This design is not simply to take a “compromise” porosity as a whole, but to let different areas of the implant undertake mechanical and biological functions respectively. This bionic thinking shows that the relationship between porosity and elastic modulus should not only be discussed at the overall level, but also be differentiated in spatial distribution. As another key structural parameter, the pore size not only affects the elastic modulus, but also directly affects the material transport and cell behavior. Too-small pore size can improve elastic modulus, but it will hinder cell growth and vascularization. Excessive pore size may make the mechanical properties fall below the safety threshold. Mahzad Sadati et al. [76] combined Kelvin cell with other cells, and found that the stress transfer efficiency of different sibling elements was different at the contact interface, which led to a change in the overall elastic modulus. The work of Zahra Yazdanpanah et al. [77] further revealed the influence of interlayer stacking mode: The relationship curves between the elastic modulus and porosity of the lattice structure and staggered structure intersected at about 55% porosity. Below this value, the staggered structure was harder, while above this value, the lattice structure was dominant. This discovery shows that different stacking strategies should be adopted to optimize the modulus matching in low-porosity and high-porosity regions respectively.

Through precisely regulating porosity and pore size, combined with different unit cell topologies, spatial distribution and post-treatment processes, additive manufacturing technology can realize accurate design and wide-range regulation of the elastic modulus of bone implants, thus effectively matching the mechanical properties of native bones in different parts, and providing a feasible path to solve the stress shielding problem.

#### 4.1.3. Effect of Pore Characteristics on Wear Resistance of Implants

Although a porous structure is beneficial to the growth of bone tissue, the introduction of pores usually reduces the bearing capacity and surface integrity of the material, which has a noticeable impact on the wear resistance of implants. It is not simply that pores weaken the wear resistance in the field of laser additive manufacturing. Goyal et al. [78] compared the wear resistance of direct metal laser sintering (DMLS) with that of traditional casting Ti-6Al-4V alloy, and found that the wear resistance of the DMLS sample was better than that of the casting sample, although the porosity was higher. It showed that the existence of a certain number of pores under the compensation of high hardness would not directly lead to a decrease in wear resistance, and even the friction interface behavior could be improved by increasing the surface hardness. Wu et al. [79] prepared a Voronoi structure and randomized dodecahedron structure with similar porosity and average pore size but different pore structures by electron beam melting technology. The wear resistance test showed that the wear mass loss of the Voronoi structure was about 6 times higher than that of the randomized dodecahedron structure. The reason for this difference was that the Voronoi structure generated a completely closed strut network based on the Tai Sen polygon algorithm, and the structural boundary was smooth and complete. However, the stress of the open strut generated by the random structure after periodic cell scaling and boundary cutting was highly concentrated under shear friction load, and it was easy to break away. A systematic study was performed by Zhang et al. [80] on the effect of micro-arc oxidation voltage on the wear resistance of a porous coating on a titanium alloy surface. It was found that the coating prepared under the condition of 360 V had the highest volume ratio of communicating pores, and the wear debris generated during the wear process could enter the large-size communicating pores, thus forming micro-protrusions for auxiliary bearing, alleviating local stress concentration. This showed that for the surface porous coating, the improvement of wear resistance depended not only on the increase in thickness and hardness, but also on the formation of appropriate and interconnected pores to accommodate wear debris. In addition to improving the wear resistance by optimizing the pore structure itself, filling polymer into the metal porous structure also provides a new way to improve the wear resistance of implants. Costa et al. [81] prepared a NiTi-PEEK multi-material composite structure by selective laser melting and hot pressing. It was found that the specific wear rate of the composite structure was reduced by 82% on average compared with the pure NiTi porous structure, and the friction coefficient was also significantly reduced. The phenomenon was mainly due to the self-lubricating property and good wear resistance of PEEK, which filled the open pores of the metal skeleton, effectively blocked the generation and accumulation of wear debris, and reduced the shear stress at the contact interface. To clearly compare the effects of the above four different pore design strategies on the wear resistance, Table 4 summarizes the wear resistance differences of implants with different pore structures.

It can be seen from the above research that the influence of pore characteristics on the wear resistance of implants is not a simple negative correlation, but highly depends on the pore size distribution, connectivity, boundary integrity and whether auxiliary filling materials are introduced. Future research should further explore the quantitative relationship between pore characteristics and wear mechanism under different physiological load conditions, and provide more accurate wear resistance data for porous implants facing specific clinical needs.

#### 4.1.4. Fatigue Performance

For bone implants in vivo, it is necessary to bear periodic loads from daily activities for a long time. Fatigue performance directly determines the long-term stability and service life of implants, and it is also one of the key indexes to evaluate the success of clinical trials. Nevertheless, the porous structure not only reduces the elastic modulus, but also introduces a large number of micro-geometric features, which makes its fatigue behavior not only a simple material problem, but also a systematic problem highly dependent on structural design, manufacturing technology and post-treatment conditions.

**Figure 9:**
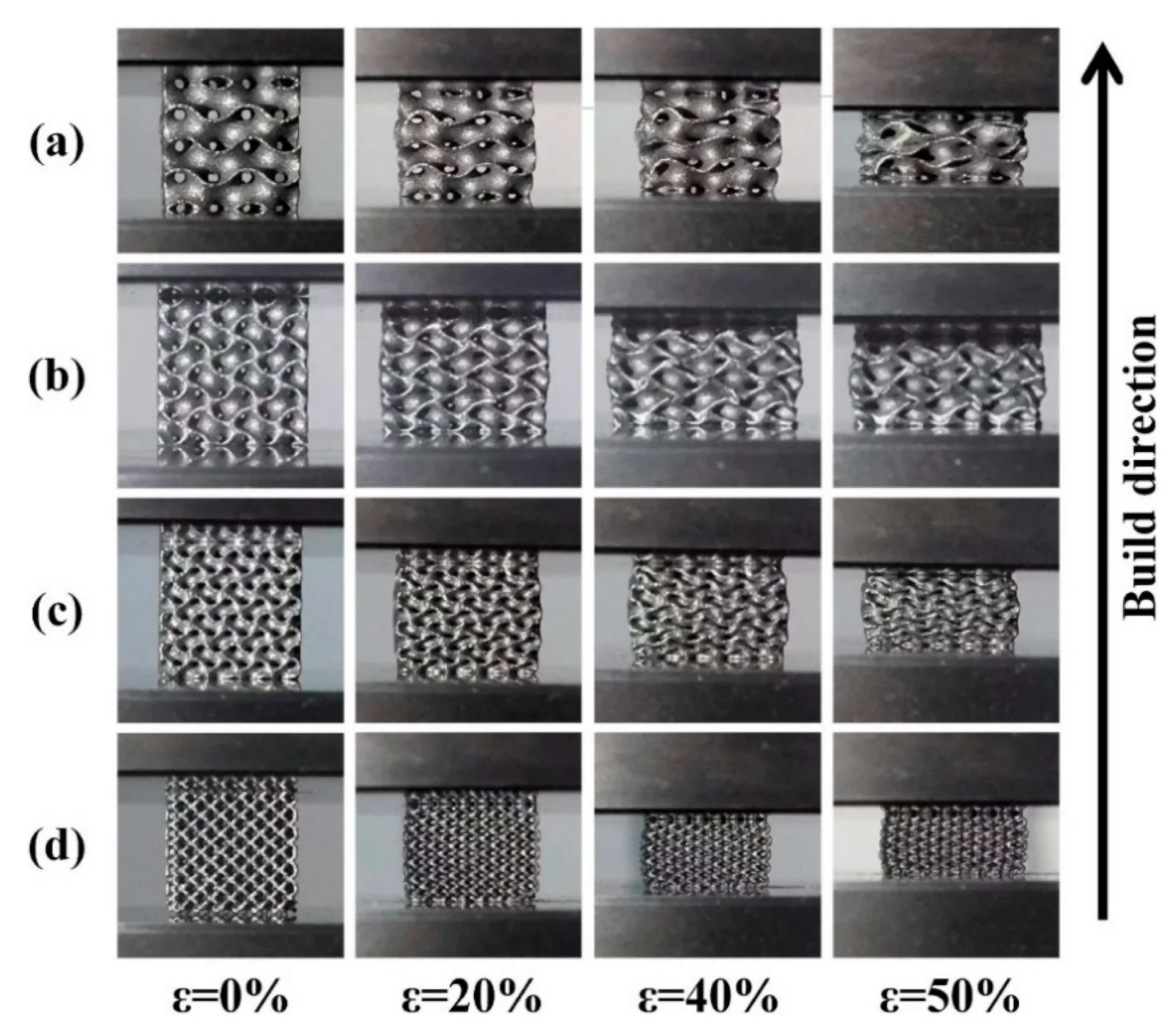
Macroscopic compression deformation images of TPMS and RDOD tantalum scaffolds under different strains. (**a**): 80% porosity TPMS. (**b**): 70% porosity TPMS. (**c**): 60% porosity TPMS. (**d**): 70% porosity RDOD [83].

Ziaie et al. [82] combined topology optimization with a TPMS structure, and systematically analyzed the mechanical compatibility of a hip stem with uniform porosity and gradient porosity. Although the uniform pore structure was better in relieving stress and shielding, its fatigue safety factor was lower than 1, and thus could not meet the requirements of clinical mechanics, while the gradient design successfully improved the safety factor to above 1. It showed that simply pursuing high porosity to reduce stiffness often comes at the expense of fatigue strength, and increasing the relative density locally in the stress concentration area through gradient design was an effective strategy to balance this contradiction. Ni et al. [83] prepared tantalum stents with a TPMS Gyroid structure and RDOD structure with a porosity of 70% by laser powder bed melting technology, and compared their differences in compression fatigue properties. The compression effects of TPMS and RDOD tantalum stents at different strain levels are shown in Figure 9. The results showed that the compressive yield strength and elastic modulus of the TPMS scaffold with 70% porosity were significantly better than those of RDOD and traditional truss structures, and there was no fatigue failure after 10^6^ cycles of loading, while the RDOD scaffold had fatigue fracture due to stress concentration at the joints. This shows that the TPMS porous tantalum scaffold has excellent anti-fatigue damage ability under cyclic loading, which provided an experimental basis for anti-fatigue design of bone implants. Kazemivand et al. [84] studied three configurations of Diamond, FCC and Gyroid at the same time, and found that there were obvious differences in fatigue behavior even within TPMS. The Gyroid structure showed the longest fatigue life under the same relative density because its mean curvature was close to zero and its stress distribution was the most uniform. The study also found that after the fatigue data of different geometries were normalized by their yield stress, the fatigue data of lattice structures with different geometries and relative densities all converged to a main curve, which indicated that their fatigue response mainly depended on their quasi-static yield strength. Meanwhile, it can be seen that the fatigue life of complex porous structures can be accurately predicted only by relatively simple quasi-static compression tests. Dallago et al. [85] found that there was obvious deviation between the actual formed structure and the design model by scanning the regular cubic lattice formed by SLM with high-precision micron CT and reverse reconstruction. The average cross-sectional radius of the horizontal pillar was more than 50% larger than the design value, accompanied by obvious axis deviation and corrugated morphology. The study also found that hot isostatic pressing treatment could effectively eliminate internal pores, but it could not improve surface defects, so the improvement of fatigue performance was very limited. Chao et al. [86] designed a bionic porous structure based on the Voronoi principle, systematically changed the irregularity and observed its influence on fatigue performance. It was shown that when the irregularity increased from 0 to 0.5, the number of fatigue cycles dropped to one third of the former. This phenomenon shows that when pursuing structural bionics and imitating the randomness of native bones, we must pay attention to the possible decline in fatigue performance caused by excessive irregularity. In order to show the differences in fatigue performance of different structural types and their key influencing factors more intuitively, Table 5 compares several typical structures discussed above.

Many factors contribute to the fatigue performance of porous bone implants, and the TPMS structure and gradient design show great potential in mechanical compatibility and fatigue resistance. However, no matter what topology design is adopted, the inherent geometric defects, surface quality and residual stress of additive manufacturing have become bottlenecks restricting its upper limit of fatigue performance. Thus, future research not only needs to explore a better topology, but also should focus on developing high-precision manufacturing technology, effective post-processing technology and fatigue life prediction models that can accurately take manufacturing defects into consideration.

### 4.2. Effects of Structural Design on Biological Properties

#### 4.2.1. Regulation of Pore Structure on Cell Proliferation and Osteogenic Differentiation

The stability of osseointegration between the bone implant and host bone tissue is highly dependent on its internal microstructure. A complex microenvironment is composed of pore morphology, pore size, porosity and spatial distribution of pores. Pore structures are the interface between cells and implants, and their geometric characteristics largely determine the initial adhesion of cells, subsequent proliferation and the depth and quality of bone tissue growth.

Li et al. [87] fabricated a gradient porous structure imitating a Haversian system based on the TPMS method from the perspective of bionic design. Its surface curvature characteristics could effectively promote the expression of osteogenesis-related genes and proteins, and enhance alkaline phosphatase activity and mineralization level. This showed that bionic structures with a specific curvature could actively guide cells to differentiate into osteoblasts. In in vitro cell experiments, the porous structure itself could also regulate the differentiation process of osteoblasts, so that the specific combination of pore size and porosity could significantly enhance the expression of osteoblast-related genes. However, it needs to be confirmed by further research in a more complicated in vivo environment. Nevertheless, not all bionic structures can achieve the same effect. Mamuti et al. [88] compared TPMS with Voronoi structures with different irregularities, and found that there were significant differences in the regulatory effects of structural morphology on cell behavior. The results of their fluid simulation and cell experiments showed that the TPMS structure had higher permeability because of its smooth and continuous internal curved surface, which was beneficial to the transport of nutrients and cell metabolism, thus promoting cell adhesion and proliferation more effectively. Compared with the Voronoi structure, although it is closer to natural trabecular bone in morphology, with the increase in irregularity, its internal fluid channel becomes tortuous and its permeability decreases, which may further limit the uniform distribution and proliferation of cells. Zhao et al. [89] used powder metallurgy titanium scaffolds to carry out static cell culture experiments in vitro, prepared titanium scaffolds with different pore sizes and porosity by powder metallurgy, and found that pore size and porosity were inseparable factors, which jointly affected protein adsorption and cell differentiation. Furthermore, the structure with smaller pore size and higher porosity could adsorb more proteins, and promote the expression of osteogenesis-related genes and the mineralization of extracellular matrix after two weeks of cell culture. When Wang and Yu [90] compared the irregular porous Ti-6Al-4V and Ti2448 alloy scaffolds with different pore sizes, they also found that with an increase in pore size, the adhesion and proliferation ability of cells on the scaffold surface gradually increased, which was more conducive to promoting osteoblast differentiation. This also shows that pore size has an important influence on the proliferation and differentiation of cells when designing irregular porous structures. Lei et al. [91] designed two kinds of rod-like lattices, octagonal and rhombic, taking alumina ceramics as the research object. Their cell experiment results in vitro showed that the cell proliferation depended on the specific pore structure and pore size.

It is very complicated for the pore structure to regulate the biological properties of implants. Bionic gradient design can guide the deep growth of cells by simulating the natural microenvironment, and the combination of pore size and porosity in a specific range can jointly promote osteogenic differentiation. So, the design of high-performance bone implants must take into account the multidisciplinary factors such as cell behavior, fluid mechanics and materials science.

#### 4.2.2. Promotion Effect of Bionic Hierarchical Porous Structure on Osseointegration

The quality of osseointegration will directly affect the long-term stability of bone implants in vivo. Although the traditional homogeneous porous structure can promote the growth of bone tissue to a certain extent, it is often difficult to take into account the dual needs of mechanical support and biological activity induction. Therefore, it has become an important strategy to imitate the hierarchical porous structure of native tissue in the design of biomedical bone implants.

**Figure 10:**
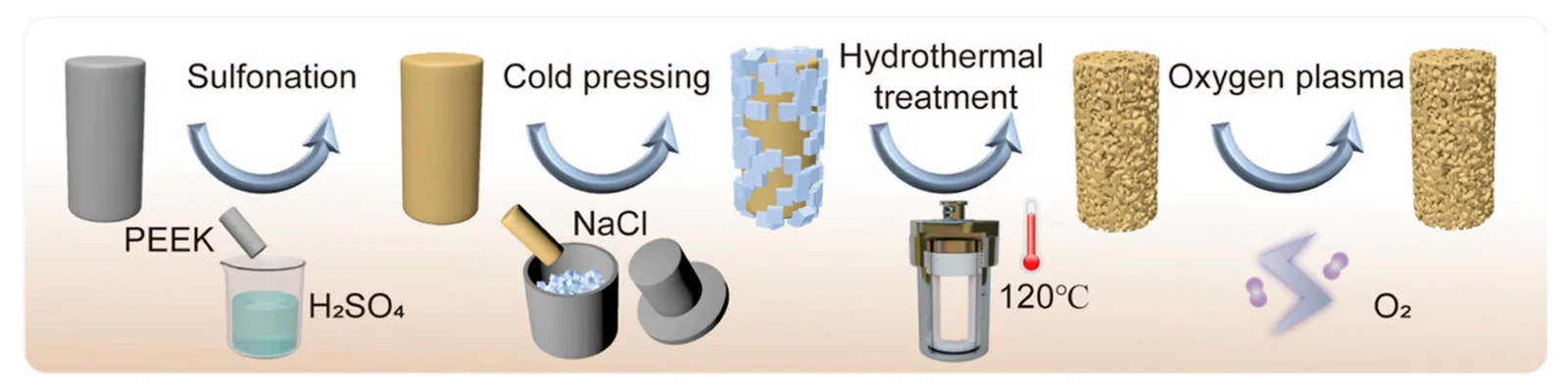
Schematic illustration of preparation of PEEK surface with 3D hierarchical porous structure.

The primary mechanism by which a bionic hierarchical porous structure promotes osseointegration is that it can simulate the microenvironment of native tissue and guide cell behavior and tissue growth. Through the combination of 3D printing technology and hydrothermal treatment, Wu et al. [92] successfully fabricated a bionic hierarchical porous structure on the surface of calcium phosphate bioceramic scaffolds. It was found that the bionic hierarchical porous structure, especially the micro–nano-whisker coating on BCP surface, could significantly improve the osseointegration ability of the bioceramic scaffold by regulating cell adhesion behavior and enhancing protein adsorption ability. Chen et al. [93] fabricated a three-dimensional hierarchical porous structure with micron-sized pores and 100-micron-sized pores on the surface of a PEEK implant by sulfonation combined with a cold-pressed pore-forming agent. The preparation method is shown in Figure 10. This structure provided three-dimensional growth space for bone marrow mesenchymal stem cells (BMSCs) without damaging the mechanical properties of the materials, and significantly improved their osteogenic differentiation ability. Lei et al. [94] broke through the limitation of machining accuracy by precisely adjusting the scanning distance and profile laser power of SLM. Connected micron-sized pores were fabricated in situ in the macro pores of the Ti-6Al-4V scaffold, which effectively reduced the elastic modulus of the scaffold to adapt to the mechanical microenvironment of osteoporosis and provided rich space for cells to attach and grow.

Inspired by the structure of natural femur and cancellous bone, Gui et al. [95] successfully prepared a kind of hydroxyapatite scaffold with a bionic gradient pore structure by using digital light processing (DLP) technology, and loaded biomaterials with different functions on the inner and outer layers respectively, thus realizing the synergistic repair of outer bone conduction and inner bone induction. This functional design in different regions enabled the central area of a large-size bone defect to get sufficient osteogenic stimulation, and realized rapid bone repair with internal and external synchronization. The advantage of a hierarchical porous structure is also reflected in its ability to actively regulate the immune microenvironment under more complicated pathological conditions. Wang et al. [96] fabricated a micron-scale network of bionic extracellular matrix (ECM) in a 3D-printed porous scaffold of Ti-6Al-4V, and wrapped Mg-MOF-74 nanoparticles in the silk fibroin network. The composite scaffolds not only provided excellent mechanical support, but also polarized macrophages from the pro-inflammatory M1 phenotype to anti-inflammatory M2 phenotype through the degradation of the SF network, thus creating an immune microenvironment conducive to bone regeneration and promoting high-quality osseointegration between implants and osteoporotic bones. In addition, the graded porous coating of TiO_2_ doped with Si/P nanocrystals prepared by Xue et al. [97] could also induce the timely transformation of macrophages from M1 to M2 after photothermal sterilization, creating a favorable immune environment for osseointegration after infection.

A bionic hierarchical porous structure not only provides space for cells to grow, but also regulates protein adsorption, cell adhesion and immune response on the micro–nano scale by simulating the hierarchical characteristics of native bones. Currently, most research is still at the observation level of structure and biological phenomena, lacking analysis of the interaction between pores of different scales. This multi-scale and multi-functional structural design strategy is becoming an important development direction to overcome the problem of osseointegration in complex clinical situations such as large-scale bone defects, osteoporosis and infection.

## 5. Discussion

In this paper, the design criteria, typical structural types and performance control paths of bone implants for additive manufacturing are presented, but there is a lack of cross-dimensional comparative analysis of multiple influencing factors to promote these designs from experimental research to clinical applications. This section focuses on the internal relationship between structural design and materials, processes and properties, focusing on the key issues facing transformation applications, such as manufacturing repeatability, sterilization adaptability and numerical prediction methods of implant corrosion, degradation and residual strength.

### 5.1. Performance Regulation in Structural Design

The design of bone implants requires finding a balance between mechanical strength and biological functionality. Current research shows that this balance is influenced by three factors—topological configuration, material system and additive manufacturing process—and there is a significant coupling relationship among them, which is not independent. Under the condition of similar porosity, the influence of topological structure on fatigue performance is particularly prominent. The TPMS structure, especially the Gyroid structure, is generally superior to the rod lattice structure in fatigue resistance under cyclic loading. The reason for this is that the TPMS structure utilizes continuous curved surfaces for the transitions, which prevents stress concentration at the rod-like joints and allows for a more even distribution of loads. By contrast, the mechanical properties of rod-like lattices are often limited by the weak links of nodes, which are prone to local bending or fracture under cyclic load.

The forming process of materials directly affects the structural accuracy and final performance of implants. Taking laser powder bed melting technology as an example, this process inevitably introduces high surface roughness, residual stress and internal pores when manufacturing metal implants. Although a rough surface is beneficial to early osteoblast adhesion, it can also easily become the source of fatigue cracks. Hot isostatic pressing can eliminate internal pores, but the improvement effect on surface defects is limited, so the fatigue life is not significantly improved. Regarding the optimal range of pore sizes, the hierarchical structure, by introducing micro- and nano-scale features within the macroscopic pores, shows that osseointegration is not determined solely by the size of the pores; it is also influenced by factors such as surface curvature, fluid shear forces, and protein adsorption behavior. This means that the aperture selection should be expanded from a single physical size problem to a multi-dimensional design problem including local geometry and biological signals.

### 5.2. Engineering Challenges for Clinical Translation

Powder residue in complex interconnected pore structures is a practical engineering problem. If the powder is not completely removed, particles may be released after implantation, which may induce an inflammatory reaction and affect the long-term stability of the implant. In the design, the channel structure convenient for powder discharge should be considered, not just from the biomechanical point of view. The influence of sterilization on the properties of materials cannot be ignored. Common sterilization methods such as high-pressure steam sterilization and gamma irradiation have little influence on the mechanical properties of a metal matrix, but for implants loaded with bioactive coatings or drug-loaded coatings, high temperature and irradiation may lead to coating degradation, drug inactivation or decreased interfacial bonding strength. Moreover, for structures with complex internal pores, whether the sterilization medium can fully penetrate into the deep pores and ensure thorough sterilization also needs special verification.

Fatigue performance is the core index to measure the long-term safety of load-bearing bone implants, but there are still obvious limitations in the fatigue research of porous structures at present. On the one hand, most fatigue tests are carried out in an atmospheric environment, and uniaxial compression cyclic loading is adopted, which is quite different from multi-axial dynamic load and corrosive physiological environments in vivo, so the test results have difficulty directly reflecting the real service state in vivo. On the other hand, the sample specifications and loading standards used in different studies are not uniform, the fatigue data obtained are not comparable, and a standardized database that can support engineering design has not yet been formed. Meanwhile, existing fatigue data have mostly been obtained based on small samples of standard size, while the stress distribution of customized complex implants is more complicated. At present, there is still a lack of fatigue life prediction methods that can simultaneously consider structural geometry, manufacturing defects and service environment.

### 5.3. Prediction Methods for the Corrosion, Degradation, and Residual Strength of Implants

Along with the rise of biodegradable metal materials such as magnesium (Mg) in the field of bone implantation, the gradual corrosion degradation and mechanical property attenuation of implants in service have become the key to determining their clinical success. The traditional experimental evaluation cycle is long, the cost is high, and it is difficult to cover the complex and changeable physiological environment in vivo. Thus, it is of great scientific and clinical significance to develop a calculation model that can describe the corrosion–mechanical coupling behavior and predict the remaining life.

The two-way coupling between corrosion and mechanics is the core scientific problem of numerical modeling of degradable implants. At present, the mainstream modeling routes are divided into two types—the phase field diffuse reflection interface method and non-local peridynamics method—which solve the problems of tracking the corrosion moving interface and multi-field coupling from different numerical paradigms. Aiming at the inherent defects of the traditional continuum model in dealing with moving boundaries and interface field discontinuity, Hermann et al. [98] proposed a nonlocal Nernst–Planck–Poisson (NNPP) corrosion modeling system. This method integrated ion diffusion, electromigration and electrochemical reactions into the nonlocal framework of near-field dynamics, and fully considered the transport and heterogeneous precipitation reaction of more than 10 ions in simulated body fluids, which was closer to the electrochemical characteristics of real physiological environments. Under the strict proof of nonlocal–local convergence, the model had both physical integrity and numerical stability, which provided a new technical path for three-dimensional corrosion simulation of implants with complex morphology. Porous bone scaffolds and other implants have special phenomena such as limited ion transport, stress concentration and local deposition of degradation products because of their complex geometric shapes. Accurate simulation of their degradation behavior is the key requirement to support clinical applications. Kovacevic et al. [99] systematically carried out a simulation study on the degradation of a porous bone scaffold and bone plate screw system: for the porous scaffolds with BCC and FCC typical lattice structures, it was found that the initial plastic strain zone introduced in the manufacturing and implantation process would become the initiation source of pitting corrosion, and the local corrosion rate was significantly higher than that in the uniform region. For the bone plate screw system, the simulation results showed that the stress concentration around the screw hole would induce local accelerated corrosion and form crack-like corrosion defects, which would eventually lead to brittle fracture of the bone plate along the cross-section between holes, directly revealing that the geometric discontinuity area was a high-risk position for the failure of degradable implants. Gaziano et al. [100] first output the geometric shapes of screws at different degradation times through the phase field model, then reconstructed them into three-dimensional solids, then carried out independent statics and fatigue analysis, and systematically calculated the equivalent fatigue stress under axial and bending load conditions. This study comprehensively considered the decline of bearing capacity caused by degradation and the accumulation of cyclic load during use, and could quantitatively give the critical failure time under different activity intensities, which can provide a reference for the formulation of postoperative rehabilitation programs.

## 6. Conclusions and Future Prospects

The rapid development of additive manufacturing technology has overcome the geometric constraints of conventional subtractive manufacturing and casting, driving the evolution of bone implant design from standardized solid configurations toward personalized, bionic architectures.

This review systematically analyzes the research progress of additively manufactured medical bone implants in terms of structural design, mechanical adaptation, biological response and performance regulation. By collating relevant research findings worldwide in recent years, this work comprehensively analyzes the performance characteristics of typical configurations, including regular porous structures, TPMS architectures, gradient composite structures and biomimetic natural structures. It also discusses the application potential of design methodologies—based on CT/MRI image reconstruction, topology optimization and additive manufacturing—in the development of patient-specific bone implants.

In terms of mechanical adaptation, the elastic modulus mismatch between conventional dense metallic implants and human cortical bone represents the primary cause of stress shielding and implant instability. Mechanical properties of native bones in different parts can be effectively matched by accurately adjusting porosity, pore size and equivalent elastic modulus of porous structures.

In the aspect of biological performance, the regulation of cellular behavior by porous structure depends not only on pore size and porosity, but also on surface curvature, fluid shear stress and the immune microenvironment. Biomimetic hierarchical porous structures have been shown to guide deep cell infiltration and promote osseointegration via the incorporation of bioactive factors or immunomodulatory ions.

In addition, bionic gradient design is an important development direction of bone implant structural design. There is a continuous gradient of porosity and mechanical properties from dense cortical bone to porous cancellous bone, whereas conventional homogeneous porous structures fail to simulate this gradual transition. Functionally graded design can match the mechanical properties and biological functions of implants with the gradient characteristics of native bones by continuously changing structural parameters in space. Moreover, under the same average porosity, the elastic modulus and compressive strength of radial gradient scaffolds are significantly better than those of homogeneous porous scaffolds, and this advantage is more obvious when the average porosity is higher.

Although many breakthroughs have been made in research on bone implants for additive manufacturing, there are still many challenges from experimental research to clinical application. Based on the above analysis, future research directions can focus on the following aspects:

(1) Currently, most research focuses on the optimization of single performance. In the future, artificial intelligence and machine learning should be combined to screen the optimal structure with mechanical properties, biological activity and manufacturability in a complex design environment, so that implants can simulate the complex architecture and physiological functions of native bones more accurately.
(2) Most existing studies are based on static or quasi-static mechanical analysis. However, osseointegration and bone remodeling are inherently dynamic processes. As new bone tissue gradually infiltrates the porous structure, the overall mechanical environment and load transfer pathways between the implant and the host bone change continuously, even though the geometry and properties of the non-degradable metallic implant itself remain unchanged. Dynamic biomechanical models should be developed in the future. By simulating the complex dynamic mechanical environment and biological process in vivo, how the porous structure parameters dynamically affect the mechanical microenvironment, fluid flow and cell response at different healing stages should be studied.
(3) Inherent defects in the additive manufacturing process are still the key factors restricting the performance of implants. In the future, we should continue to promote the innovation of additive manufacturing equipment and technology, improve printing accuracy and surface quality, and reduce internal defects. Meanwhile, the additive manufacturing technology of new biocompatible materials and composite materials should be explored to expand the application scope of implants.

## Figures and Tables

**Table 1:** The key constraints and post-treatment effect of additive manufacturing process adaptation.

Forming Type	Representative Technology	Process Principle	Applicable Materials	Process Advantages	Process Disadvantages
Vat Photopolymerization	DLP	The surface is exposed and solidified, and the digital element controls the light path to project layer by layer	Photosensitive resin/ceramic paste	High resolution and high molding speed	The forming size is limited, and it is difficult to manufacture large-size implants
Material Extrusion	FDM, DIW	The material is melted or squeezed, and then stacked layer by layer through the nozzle	Thermoplastic polymers, hydrogels	Flexible material selection and low cost	The nozzle diameter limits the accuracy and the step effect affects the surface quality
Material Jetting	Polyjet, SLA	After the granular material is sprayed, it is cured by ultraviolet light or scanned by laser point by point	Photosensitive resin, ceramics	High printing accuracy and excellent surface quality	Uncured photosensitive resin is toxic
Powder Bed Fusion	3DP, SLS, SLM	After the powder is leveled, the adhesive is sprayed or selectively melted by a laser	Metals, polymers, ceramics	High material utilization rate and strong ability to manufacture complex structures	High temperature causes materials to deform easily

**Table 2:** Comparison of porous structures of four types of bone implants.

	Regular Porous Structure	TPMS	Gradient and Composite Structure	Bionic Natural Structure
Representative structure	Cubes, hexahedrons, rhombic dodecahedrons, octahedrons and columnar octahedrons	Gyroid, Diamond, Primitive, Neovius, Fisher–Koch S	Radial gradient, layered gradient, tension gradient, BCC gradient, PEEK/HA composite gradient	Voronoi irregular network, quasi-uniform heterogeneous structure, natural trabecular topological structure
Fatigue performance	Stress concentration exists in the joint of the bracket, and the fatigue life suddenly drops under high porosity.	Without sharp joints, Gyroid has the best high-cycle fatigue strength.	Local stress concentration may occur in the gradient transition zone.	The higher the porosity, the worse the fatigue performance.
Osteogenic differentiation induction ability	Relying on surface modification to improve cell adhesion	Curvature of curved surface can regulate the expression of osteogenic genes.	Hierarchical pore realizes layered bone conduction	Bionic morphology is close to natural cancellous bone, but insufficient penetration limits deep osteogenesis.
Difficulties in additive manufacturing	With few supports, the powder is easy to clean.	Need a lot of support, complex post-processing.	High risk of printing deformation	The error is large and the geometric accuracy is difficult to control.
Core inherent defects	It is not matched with the mechanical gradient of natural bone, and it is easy to produce interfacial stress shielding.	Excessive permeability reduces wall shear stress and inhibits early cell adhesion; some structures have high risk of printing failure.	Gradient transition zone easily produces local stress concentration, and there is no unified optimization algorithm.	Fatigue life is significantly lower than that of regular structures.
Applicable to clinical scenarios	Lower-load-bearing parts: skull, metacarpal and small bone defects.	High-load-bearing parts: spinal fusion cage, hip prosthesis and screw fixation parts.	Large weight-bearing bone defects: segmental defects of tibia, proximal femur and tumor.	Non-load-bearing bionic prosthesis: maxillofacial region
Existing research disputes	There is no unified predictive model for the variation in unit modulus at the same porosity.	Molecular mechanism of surface curvature regulating osteogenesis is not clear.	Mechanical response of gradient structure under dynamic load is lack of in vivo verification.	The optimal interval of heterogeneity is controversial.

**Table 3:** Multi-dimensional comparison of mainstream design methods of bone implants.

	Anatomical Adaptation Design Based on CT/MRI Image Reconstruction	Topology Optimization Design	Design Based on Additive Manufacturing
Core design objectives	Matching the patient’s personalized anatomical shape and recreating the stiffness distribution of primary bone	Minimizing structural flexibility, lightening and relieving stress shielding under a given load.	Transforming the theoretically optimal structure into a printable and applicable implant in reality.
Mechanical optimization ability	Only mapping elastic modulus based on static HU value, ignoring dynamic fatigue load.	The force conduction path can be directionally optimized to reduce the concentrated stress of the implant.	Without independent mechanical optimization ability, it is only used as the first two design methods of constraint superposition.
Bionic level	Completely matches the patient’s anatomical data.	Optimized structure is the mathematical optimal solution, and there is no bionic feature of natural bone.	Without bionic properties, only geometric manufacturing defects are corrected.
Calculation cost	The image reconstruction software is mature and the operation speed is fast.	Large-scale models require high computational power.	Moderate calculation cost
Inherent limitation	Limited regulatory capacity at the microstructure level.	Large numbers of overhanging thin walls are generated, and it is impossible to print the detailed features of complex structures directly.	Using it alone cannot optimize the mechanical and biological properties, and it is only an auxiliary correction method.
Difficulty in clinical implementation	Preoperative osteo-guide plate and small prosthesis have been used in clinic.	Only laboratory research, lack of standardized process adaptation scheme.	Coupling relationship between process parameters and structure cannot be unified in the existing database.

**Table 4:** Comparison of wear resistance of different porous structures.

Structural Type	Critical Features	Advantages and Disadvantages	Mechanism Analysis	Core Enlightenment
Voronoi structure	Boundary closed, no free end	Complete boundary, excellent wear resistance; the design algorithm is relatively complex	The closed boundary effectively inhibits the stress concentration under shear load, and the wear mechanism is mainly superficial abrasive wear.	Closed-loop boundary design is the primary guarantee for wear resistance of metal porous structures.
Randomized dodecahedron	Open boundary, multiple free ends	Simple modeling and easy parametric adjustment; the border is weak and badly worn	Wear mechanism is mainly fatigue brittle peeling, and the rate of debris generation increases exponentially.	Although introducing irregularity blindly is beneficial to bone growth, it will seriously damage wear resistance.
Single NiTi structure	No filling	NiTi is superelastic; higher wear rate	Wearing mechanism is a mixed mode of adhesive wear and abrasive wear, and abrasive debris cannot be effectively contained.	Super-elasticity of a single material cannot fundamentally solve the problem of weak shear resistance of porous structures.
NiTi-PEEK composite structure	PEEK filling	Greatly improve the wear resistance and reduce the friction coefficient; the interfacial bonding strength is not clear	PEEK’s self-lubricating characteristics block the generation and accumulation of debris.	Multi-material strategy can greatly improve the wear resistance, but the long-term stability of the interface is doubtful.

**Table 5:** Comparison of fatigue performance of different structural types.

Structural Type	Materials	Fatigue Performance	Advantages	Disadvantages
Voronoi rule structure	Ti-6Al-4V	Fatigue cycle times are three times that of irregular structures	Fatigue life is significantly better than that of irregular structures	Lack of bionics, which is quite different from native morphology
Voronoi irregular structure	Ti-6Al-4V	Minimum fatigue life	Good bionics, the shape is close to native bones	Irregularity leads to stress concentration and poor fatigue performance
Cubic lattice	Ti-6Al-4V	2.06 MPa (10^6^ cycles)	Mature process and easy manufacture	Measured fatigue strength is only 38% of the ideal prediction
Cubic lattice (HIPed)	Ti-6Al-4V	2.02 MPa (10^6^ cycles)	HIP can eliminate internal pores	Surface defects are not improved, and fatigue performance is not improved
Gyroid (70% porosity)	Tantalum (Ta)	No fatigue failure under 0.8σ yield stress (10^6^ cycles)	Smooth surface avoids stress concentration and has excellent fatigue performance	High material cost and high density
RDOD (70% porosity)	Tantalum (Ta)	Fatigue fracture at 26 MPa (10^6^ cycles)	Simple structure and easy design	Stress concentration at the joints, low fatigue strength

## Data Availability

The manuscript does not associate with any external data.

## Abbreviations

The following abbreviations are used in this manuscript:

AM	Additive manufacturing
TPMS	Triply Periodic Minimal Surface
TPMS-D	Triply Periodic Minimal Surface-Diamond
PEEK	Polyether Ether Ketone
β-TCP	β-tricalcium phosphate
SLA	Stereolithography
CT	Computed Tomography
DMLS	Direct Metal Laser Sintering
VIKOR	VIseKriterijumska Optimizacija I Kompromisno Resenje
ASTM	American Society for Testing and Materials
DLP	Digital Light Processing
FDM	Fused Deposition Modeling
DIW	Direct Ink Writing
3DP	Three-Dimension Printing
SLS	Selective Laser Sintering
SLM	Selective Laser Melting
MG-63	MG-63 human osteosarcoma cells
FEM	Finite Element Method
CFD	Computational Fluid Dynamics
ALP	Alkaline Phosphatase
OPN	Osteopontin
HA	Hydroxyapatite
MRI	Magnetic Resonance Imaging
CAD	Computer-Aided Design
LPBF	Laser Powder Bed Fusion
RDOD	Rhombic dodecahedron
FCC	Face-centered cubic
BCP	Biphasic calcium phosphate
BMSCs	Bone marrow mesenchymal stem cells
ECM	Extracellular matrix
Si	Silicon
P	Phosphorus
TiO_2_	Titanium dioxide
